# Different Roles of Mitochondrial Calcium Uniporter Complex Subunits in Growth and Infectivity of *Trypanosoma cruzi*

**DOI:** 10.1128/mBio.00574-17

**Published:** 2017-05-09

**Authors:** Miguel A. Chiurillo, Noelia Lander, Mayara S. Bertolini, Melissa Storey, Anibal E. Vercesi, Roberto Docampo

**Affiliations:** a Departamento de Patología Clínica, Universidade Estadual de Campinas, Campinas, São Paulo, Brazil; b Center for Tropical and Emerging Global Diseases and Department of Cellular Biology, University of Georgia, Athens, Georgia, USA; Washington University School of Medicine

**Keywords:** calcium signaling, mitochondria, *Trypanosoma*, uniporter

## Abstract

*Trypanosoma cruzi* is the agent of Chagas disease, and the finding that this parasite possesses a mitochondrial calcium uniporter (TcMCU) with characteristics similar to that of mammalian mitochondria was fundamental for the discovery of the molecular nature of MCU in eukaryotes. We report here that ablation of *TcMCU*, or its paralog *TcMCUb*, by clustered regularly interspaced short palindromic repeat (CRISPR)/Cas9 led to a marked decrease in mitochondrial Ca^2+^ uptake without affecting the membrane potential of these cells, whereas overexpression of each gene caused a significant increase in the ability of mitochondria to accumulate Ca^2+^. While *TcMCU-*knockout (KO) epimastigotes were viable and able to differentiate into trypomastigotes, infect host cells, and replicate normally, ablation of *TcMCUb* resulted in epimastigotes having an important growth defect, lower rates of respiration and metacyclogenesis, more pronounced autophagy changes under starvation, and significantly reduced infectivity. Overexpression of *TcMCUb*, in contrast to what was proposed for its mammalian ortholog, did not result in a dominant negative effect on TcMCU.

## IMPORTANCE

The finding of a mitochondrial calcium uniporter (MCU) in *Trypanosoma cruzi* was essential for the discovery of the molecular nature of this transporter in mammals. In this work, we used the CRISPR/Cas9 technique that we recently developed for *T. cruzi* to knock out two components of the uniporter: MCU, the pore subunit, and MCUb, which was proposed as a negative regulator of MCU in human cells. In contrast to what occurs in human cells, MCU is not essential, while MCUb is essential for growth, differentiation, and infectivity; has a bioenergetic role; and does not act as a dominant negative subunit of MCU.

## INTRODUCTION

*Trypanosoma cruzi* is the etiologic agent of Chagas disease, an enormous burden on human health in the American continent, which has four major developmental stages that alternate between an insect vector and a mammalian host. Two are replicative forms, the epimastigote found in the insect vector intestine and the intracellular mammalian form or amastigote, and two are nonreplicative, the metacyclic trypomastigote found in the rectum and urine of the vector and the bloodstream trypomastigote found in the mammalian host. All these forms appear to have functional mitochondria with an active oxidative metabolism (1, 2).

The finding that mitochondrial Ca^2+^ transport in *T. cruzi* is electrogenic, has low affinity and high capacity, and is inhibited by ruthenium red, as occurs with vertebrate mitochondria, identified the presence of a mitochondrial calcium uniporter (MCU) in trypanosomatids (3, 4). The presence of MCU in trypanosomes together with its absence in yeast (5) led to the identification, first, of the gene encoding an MCU modulator (mitochondrial calcium uptake 1 [MICU1]) (6) and then of the gene encoding the MCU of mammalian cells (7–9). In recent work, we demonstrated that the MCU of *Trypanosoma brucei*, which belongs to the group of parasites that produce African trypanosomiasis or sleeping sickness, is essential for the regulation of the bioenergetics of the parasite and its growth and infectivity (10).

After the discovery of the molecular identity of MCU, other components of the MCU complex were described (11, 12). One of them, MCUb, was shown to exert a dominant negative effect in HeLa cell mitochondria, reducing the mitochondrial Ca^2+^ increase evoked by agonist stimulation (13).

In this work, we report that knockout of the *T. cruzi MCU* (*TcMCU*) gene by clustered regularly interspaced short palindromic repeat (CRISPR)/Cas9 in *T. cruzi* epimastigotes (14) abolishes mitochondrial calcium uptake without affecting their mitochondrial membrane potential (ΔΨ_m_) and reduces growth in low-glucose medium. However, epimastigotes conserve their ability to differentiate into metacyclic trypomastigotes and infect mammalian cells. In contrast to a previous report on HeLa cells (13), overexpression of *TcMCUb* does not have a dominant negative effect but increases mitochondrial Ca^2+^ uptake without affecting the ΔΨ_m_. Knockout of *TcMCUb* by CRISPR/Cas9 abolishes mitochondrial Ca^2+^ transport, reduces respiration, has a significant effect on epimastigote growth, and increases autophagy. These cells have a reduced ability to differentiate into metacyclic trypomastigotes and are unable to efficiently infect cells, underscoring the relevance of TcMCUb for the parasite life cycle.

## RESULTS

### Ca^2+^ uptake by *TcMCU* and *TcMCUb* knockouts.

After the recent characterization of MCU as the channel-forming subunit of the mitochondrial calcium uniporter complex (15), several pore regulators were reported, among them mitochondrial calcium uptake 1 (MICU1), MICU2, MCUb, essential MCU regulator (EMRE), and MCU regulator 1 (MCUR1) (11). It has been suggested that MCUb is a dominant negative regulator of the uniporter complex (13). However, evidence of its influence on MCU regulation is lacking. MCUb has been identified in the *T. cruzi* genome, and similarly to its paralog MCU, it has two predicted transmembrane domains (16). Therefore, we aimed at investigating the effect of downregulation and overexpression of *TcMCUb* on the physiological role of the MCU complex in *T. cruzi*.

**FIG 1:**
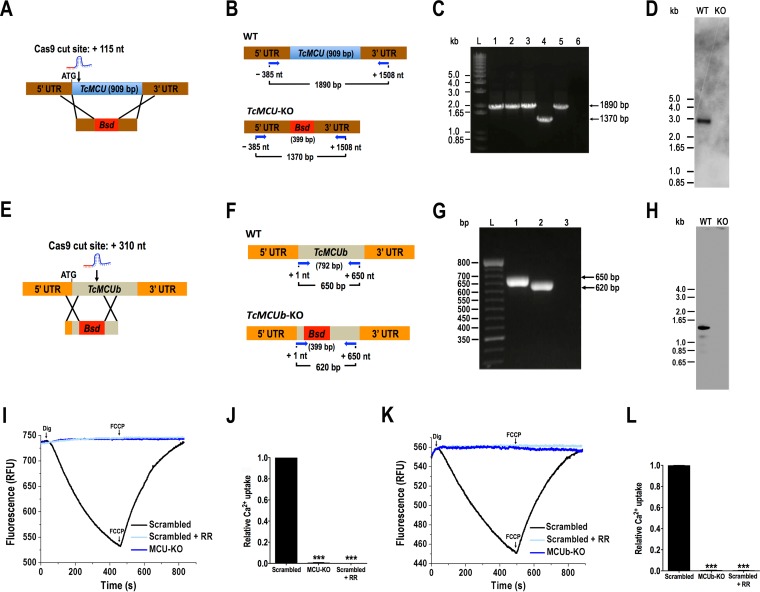
Ca^2+^ uptake by *TcMCU* and *TcMCUb* knockouts. (A) Schematic representation of the strategy used to generate a *TcMCU-*KO mutant by CRISPR/Cas9-induced homologous recombination. A double-stranded gDNA break was produced by Cas9 at nt +115 of the *TcMCU* ORF. DNA was repaired with a blasticidin *S*-deaminase (*Bsd*) cassette containing ~500-bp homologous regions from the *TcMCU* locus. (B) Primers (arrows) that were used to verify gene disruption by PCR. The intact locus generates a PCR product of 1,890 bp, while the replaced locus generates a fragment of 1,370 bp. (C) *TcMCU* was ablated at its genomic locus and replaced in genomic DNA of the KO cell line. Lanes: L, 1-kb plus ladder; 1, wild type; 2, pTREX-n control cells; 3, Cas9/pTREX-n control cells; 4, *TcMCU-*KO mutant cell line; 5, scrambled sgRNA/Cas9/pTREX-n control cells; 6, PCR negative control. (D) Southern blot analysis of wild-type (WT) and *TcMCU*-KO (KO) epimastigotes. The blot was hybridized with a 100-bp radiolabeled region of *TcMCU*. (E) Schematic representation of the strategy used to generate a *TcMCUb-*KO mutant by CRISPR/Cas9-induced homologous recombination. A double-stranded gDNA break was produced by Cas9 at nt +310 of the *TcMCUb* ORF (792 bp). DNA was repaired with a blasticidin *S*-deaminase (*Bsd*) cassette containing 100-bp homologous regions spanning from nt −20 to +80 and from nt +510 to +610 of the *TcMCUb* locus. (F) Primers (arrows) used to verify gene disruption by PCR. The intact locus generates a PCR product of 650 bp, while the disrupted locus generates a fragment of 620 bp. (G) *TcMCUb* was disrupted at its genomic locus where the *Bsd* gene replaced a 430-bp fragment in the KO cell line. Lanes: L, 1-kb plus ladder; 1, wild type; 2, *TcMCUb-*KO mutant cell line; 3, PCR negative control. (H) Southern blot analysis of wild-type (WT) and *TcMCUb*-KO (KO) epimastigotes. The blot was hybridized with a biotin-labeled probe corresponding to 430 bp of *TcMCUb* (nt +80 to +510). (I) Ca^2+^ uptake by digitonin-permeabilized epimastigotes in relative fluorescence units (RFU). MCU-KO, *TcMCU-*KO cells; Scrambled, scrambled control cells in absence or presence (+ RR) of 5 µM ruthenium red. The reaction was started after adding 50 µM digitonin in the presence of 20 µM CaCl_2_. Where indicated, 4 µM FCCP was added. (J) Quantification of data in panel I. Relative Ca^2+^ uptake at 400 s compared with epimastigotes transfected with scrambled control. (K) Ca^2+^ uptake by digitonin-permeabilized epimastigotes in relative fluorescence units (RFU). MCUb-KO, *TcMCU-*KO cells; Scrambled, scrambled control cells in absence or presence (+ RR) of 5 µM ruthenium red. The reaction was started after adding 50 µM digitonin in the presence of 20 µM CaCl_2_. Where indicated, 4 µM FCCP was added. (L) Quantification of data in panel K. Relative Ca^2+^ uptake at 400 s compared with epimastigotes transfected with scrambled control. Values in panels J and L are means ± SD (*n* = 3). ***, *P* < 0.001.

We used the recently developed CRISPR/Cas9 system with a DNA donor cassette for DNA repair (14) to knock out *TcMCU* (Fig. 1A and B) and *TbMCUb* (Fig. 1E and F) in epimastigotes. After 5 to 6 weeks of selection under G418 and blasticidin, we obtained resistant populations of epimastigotes transfected with specific single guide RNAs (sgRNAs) and blasticidin cassettes. These were constructed with long (~500-bp) flanking untranslated regions (UTRs) cloned in pGEM-T Easy vector to obtain *TcMCU-*KO cells or using long oligonucleotides (ultramers) with shorter homology regions (100 bp) to obtain the *TcMCUb-*KO cell line. Primers (see Table S1 in the supplemental material) were used in PCR experiments to check *TcMCU* and *TcMCUb* disruption in G418/blasticidin-resistant cells. As shown in Fig. 1C, *TcMCU* was ablated and replaced by the blasticidin resistance gene in *TcMCU*-KO cells. Disruption of the *TcMCUb* gene was also demonstrated using specific primers (Fig. 1G). In addition, Southern blot analyses demonstrated that *TcMCU* (Fig. 1D) and *TcMCUb* (Fig. 1H) were absent in genomic DNA of the KO cell lines.

10.1128/mBio.00574-17.7 TABLE S1  Oligonucleotides used in this work. Download TABLE S1, DOCX file, 0.1 MB. Copyright © 2017 Chiurillo et al. 2017 Chiurillo et al. This content is distributed under the terms of the Creative Commons Attribution 4.0 International license.

To determine the capacity of *TcMCU-*KO and *TcMCUb-*KO cell lines to take up Ca^2+^, we monitored Ca^2+^ uptake with calcium green-5N probe in digitonin-permeabilized epimastigotes. A decrease in fluorescence indicates decreasing medium Ca^2+^ or increasing vesicular Ca^2+^. Figures 1I and K show that addition of 50 µM digitonin in the presence of 5 mM succinate and 20 µM Ca^2+^ produced a fast decrease in Ca^2+^ concentration starting after a period of about 60 s, which continued until addition of the uncoupler *p*-trifluoromethoxyphenylhydrazone (FCCP), which released the mitochondrial Ca^2+^ taken up. This Ca^2+^-transporting activity was fully eliminated by the addition of 5 µM ruthenium red, indicating that it is due to the uniporter. Knockout of *TcMCU* (Fig. 1I and J) or *TcMCUb* (Fig. 1K and L) abolished the *T. cruzi* mitochondrial ability to take up Ca^2+^.

### Coimmunoprecipitation of *TcMCU* and *TcMCUb* and Ca^2+^ uptake by *TcMCU-* and *TcMCUb*-overexpressing cells.

**FIG 2:**
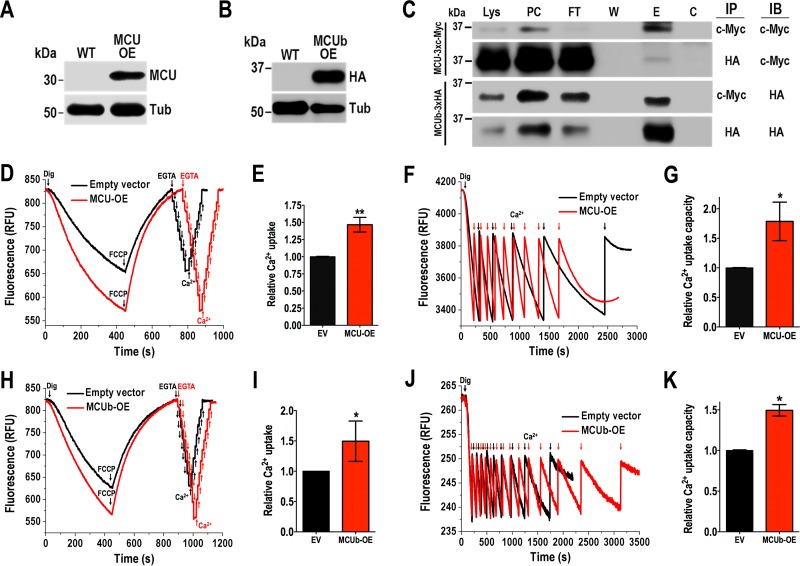
Coimmunoprecipitation of TcMCU and TcMCUb and Ca^2+^ uptake by *TcMCU-* and *TcMCUb*-overexpressing cells. (A) Western blot analysis of TcMCU in total protein extracts of wild-type (WT) and *TcMCU-*OE (MCU-OE) epimastigotes, using anti-TbMCU antibodies. Anti-α-tubulin antibodies were used as a loading control. (B) Western blot analysis of total protein extracts of wild-type (WT) and *TcMCUb*-3×HA-OE (MCUb OE) epimastigotes using anti-HA antibodies. Anti-α-tubulin antibodies were used as a loading control. (C) Anti-c-Myc and anti-HA immunoprecipitations (IP) using lysates from epimastigotes overexpressing TcMCUb-3×HA and TcMCU-3×c-Myc. Immunoblot assays (IB) were done with anti-c-Myc and anti-HA antibodies. Analyzed fractions were as follows: Lys, total lysate; PC, precleared lysate; FT, flowthrough; W, last wash; E, eluate; C, control agarose. (D) Ca^2+^ uptake by digitonin-permeabilized empty vector (pTREX-n) and *TcMCU*-OE (MCU-OE) cells. Other additions were as in Fig. 1I. EGTA (4 µM) and Ca^2+^ (2 µM pulses) were added where indicated. (E) Quantification of data in panel D. Relative Ca^2+^ uptake at 400 s compared with empty vector (EV). (F) Similar conditions as in panel D, except that reaction buffer contained 0.2% bovine serum albumin and further CaCl_2_ pulses (6 µM each time) were added to empty vector and MCU-OE cells to show the high mitochondrial capacity to take up Ca^2+^. (G) Quantification of data in panel F. Relative Ca^2+^ uptake capacity (number of Ca^2+^ pulses) compared with that of empty vector cells. (H) Ca^2+^ uptake by digitonin-permeabilized empty vector (pTREX-n) and *MCUb*-OE cells. Other additions were as in Fig. 1K. EGTA (4 µM) and Ca^2+^ (2 µM pulses) were added where indicated. (I) Quantification of data in panel H. Relative Ca^2+^ uptake at 400 s compared with empty vector. (J) Similar conditions as in panel H except that reaction buffer contained 0.2% bovine serum albumin and further CaCl_2_ additions (6 µM each time) were added to empty vector and *MCUb*-OE cells to show the high mitochondrial capacity to take up Ca^2+^. (K) Quantification of data in panel J. Relative Ca^2+^ uptake capacity (number of Ca^2+^ pulses) compared with that of empty vector cells. Values in panels E, G, I, and K are means ± SD (*n* = 3). *, *P* < 0.05; **, *P* < 0.01.

We also generated cell lines overexpressing *TcMCU* (*TcMCU-*OE) or *TcMCUb* (*TcMCUb-*OE) cloned in pTREX-n vector, as described in Materials and Methods. We recently demonstrated that endogenously tagged TcMCU localizes to the mitochondria of *T. cruzi* (17). TcMCU-OE protein also localized to the mitochondria, as demonstrated by immunofluorescence microscopy (see Fig. S1A). Antibody to *T. brucei* MCU (10) colocalized with antibody against the voltage-dependent anion channel (TbVDAC), which is a major protein of the outer mitochondrial membrane of eukaryotes. Western blot analysis detected a band of the expected size (32 kDa) in the overexpressing cell line (Fig. 2A). The band was not visible in wild-type (WT) cells, probably as a consequence of its low expression levels. *TcMCU*-OE and *TcMCUb*-OE (Fig. S1B) cells exhibited the same growth rate as control cells transfected with the pTREX-n empty vector. Western blot analysis of *TcMCUb*-OE cells using antibodies against its hemagglutinin (HA) tag confirmed its expression (Fig. 2B), and immunofluorescence analysis demonstrated its mitochondrial localization (Fig. S1C).

10.1128/mBio.00574-17.2 FIG S1  Localization and growth of *TcMCU*-OE and *TcMCUb*-OE epimastigotes. (A) Fluorescence microscopy of *TcMCU-*OE epimastigotes (MCU) using anti-TbMCU antibodies (green) and anti-TbVDAC (VDAC) antibodies as a mitochondrial marker (red). Nuclei and kinetoplast were labeled with DAPI (blue). Colocalization of TcMCU-OE and TcVDAC is shown in yellow (merge). A differential interference contrast (DIC) image is shown in the left panel. (B) Growth curve of *TcMCU-*OE (MCU-OE) epimastigotes, *TcMCUb-*OE (MCUb-OE) epimastigotes, and epimastigotes transfected with pTREX-n empty vector. (C) Colocalization of TcMCUb-3×HA-OE using anti-HA antibodies (HA) with MitoTracker (MT). (D) Western blot analysis of WT, TcMCU-3×c-Myc, and TcMCU-3×c-Myc/pTREX-n-TcMCUb-3×HA cell lines. Anti-c-Myc and anti-HA antibodies detect TcMCU-3×c-Myc (predicted size, 35 kDa) and TcMCUb-3×HA (predicted size, 31 kDa), respectively, whereas both bands are absent in WT parasites. Anti-α-tubulin antibody was used as a loading control. The TcMCU-3×c-Myc cell line was obtained by CRISPR/Cas9-mediated endogenous C-terminal tagging as described previously (15). Antibodies are indicated on the right side of the blots, and molecular weights are on the left side. There was no significant difference in intensity of bands corresponding to TcMCU-3×c-Myc detected with anti-c-Myc antibodies between TcMCU-3×c-Myc and TcMCU-3×c-Myc/pTREX-n-TcMCUb-3×HA cell lines. Bars in panels A and C, 10 µm. Values in panel B are means ± SD (*n* = 3; no significant difference). Download FIG S1, TIF file, 1.4 MB. Copyright © 2017 Chiurillo et al. 2017 Chiurillo et al. This content is distributed under the terms of the Creative Commons Attribution 4.0 International license.

To investigate whether TcMCU and TcMCUb could form oligomers as previously described with their orthologs in HeLa cells (13), we used a *T. cruzi* cell line that coexpresses the c-Myc-tagged TcMCU (TcMCU-3×c-Myc) and the HA-tagged TcMCUb (TcMCUb-3×HA), as described in Materials and Methods. Cells were lysed, and immunoprecipitation was done with either anti-c-Myc or anti-HA antibody. Western blot analyses showed that the anti-HA antibody immunoprecipitated TcMCU-3×c-Myc and the anti-c-Myc antibody immunoprecipitated TcMCUb-3×HA, revealing the MCU-MCUb interaction *in situ* (Fig. 2C).

*TcMCU*-OE (Fig. 2D and E) and *TcMCUb-*OE (Fig. 2H and I) epimastigotes showed increased Ca^2+^ uptake. The mitochondria of permeabilized control epimastigotes were capable of buffering multiple pulses of exogenously added Ca^2+^, and overexpression of *TcMCU* (Fig. 2F and G) or *TcMCUb* (Fig. 2J and K) increased significantly the ability of their mitochondria to accumulate Ca^2+^ in response to Ca^2+^ pulses. Overexpression of *TcMCUb* in epimastigotes possessing endogenously tagged TcMCU did not induce its overexpression (Fig. S1D).

### Analysis of the mitochondrial membrane potential (ΔΨ_m_) of mutant cell lines.

Safranin O was used to measure ΔΨ_m_ in digitonin-permeabilized epimastigotes in the presence of succinate as the mitochondrial substrate. To be certain that the ΔΨ_m_ was not affected in the mutants, we calibrated the ΔΨ_m_ using valinomycin and potassium as we described before (18) and expressed the changes in millivolts instead of fluorescence arbitrary units. The magnitude of this membrane potential of epimastigote mitochondria respiring on succinate was 177 ± 2.0, 179.6 ± 2.8, 177.6 ± 2.3, 177.7 ± 1.6, and 177.9 ± 1.7 mV for wild-type, *TcMCU-*KO, *TcMCU-*OE, *TcMCUb*-KO, and *TcMCUb*-OE cells, respectively (*n* = 3). When using safranin O, an increase in fluorescence after addition of digitonin indicated stacking of the dye to the energized inner mitochondrial membrane (Fig. S2A to C). Addition of ADP produced the expected small decrease in membrane potential, indicating ADP phosphorylation. ΔΨ_m_ returned to its initial level after addition of the adenine nucleotide translocator inhibitor carboxyatractyloside (CAT). Addition of FCCP collapsed the membrane potential. Neither knockout nor overexpression of either *TcMCU* (Fig. S2A to C) or *TcMCUb* (Fig. S2D to F) affected the ΔΨ_m_ at the steady state or ADP phosphorylation.

10.1128/mBio.00574-17.3 FIG S2  Mitochondrial membrane potential in mutant epimastigotes (A and B). Changes in mitochondrial membrane potential (ΔΨ_m_) of digitonin-permeabilized epimastigotes as detected by changes in safranin O fluorescence in epimastigotes transfected with scrambled sgRNA/Cas9/pTREX-n (Scrambled) or *TcMCU-*KO (MCU-KO) (A) and empty vector or *TcMCU-*OE (MCU-OE) (B). Cells (5 × 10^7^) were added to the reaction buffer (2 ml) containing 0.2% bovine serum albumin, 2 mM succinate, and 5 µM safranin O. The reaction was started with 50 µM digitonin, and 250 µM ADP, 20 µM carboxyatractyloside (CAT), and 4 µM carbonyl cyanide *p*-trifluoromethoxyphenylhydrazone (FCCP) were added where indicated. (C) Quantification of changes in ΔΨ_m_ in panels A and B. (D and E) Changes in ΔΨ_m_ in digitonin-permeabilized epimastigotes as detected by changes in safranin O fluorescence in epimastigotes transfected with scrambled sgRNA/Cas9/pTREX-n (Scrambled) or *TcMCUb-*KO (MCUb-KO) (D) and empty vector or *TcMCUb-*OE (MCUb-OE) (E). Other conditions are as in panels A and B. (F) Quantification of changes in ΔΨ_m_ in panels D and E. Values in panels C and F are means ± SD (*n* = 3; no significant differences, Student’s *t* test). Download FIG S2, TIF file, 1.4 MB. Copyright © 2017 Chiurillo et al. 2017 Chiurillo et al. This content is distributed under the terms of the Creative Commons Attribution 4.0 International license.

### Growth of *TcMCU* and *TcMCUb* mutants.

**FIG 3:**
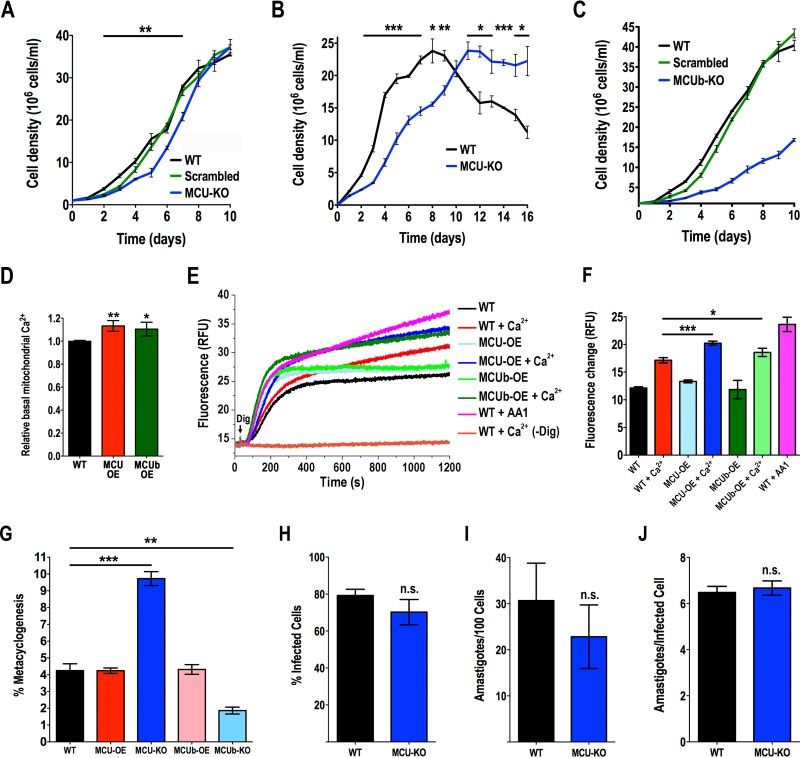
Phenotypic changes in *TcMCU* and *TcMCUb* mutant epimastigotes. (A) Growth of wild-type (WT) and *TcMCU-*KO epimastigotes (MCU-KO) and epimastigotes transfected with scrambled sgRNA (Scrambled) in LIT medium. (B) Growth of WT and MCU-KO epimastigotes in low-glucose LIT medium. (C) Growth curve of wild-type (WT) and *TcMCUb*-KO (MCUb-KO) epimastigotes and epimastigotes transfected with a scrambled sgRNA grown in LIT medium (*n* = 3). (D) Relative Rhod-2 fluorescence in control cells (WT) compared with MCU-OE or MCUb-OE cells. (E) Mitochondrial oxidative stress as measured with MitoSOX Red in control (WT), *TcMCU-*OE (MCU-OE), and *TcMCUb-*OE (MCUb-OE) epimastigotes permeabilized with 50 μM digitonin in the presence or absence of 0.5 mM Ca^2+^. Controls were treated with 2 mM antimycin A_1_ (WT + AA1) or were epimastigotes exposed to 0.5 mM Ca^2+^ in the absence of digitonin [WT + Ca^2+^ (-Dig)]. (F) Quantification of changes in panel E at 1,200 s. (G) Percentage of metacyclic trypomastigotes in epimastigote cultures after incubation in TAU 3AAG medium. Differentiation of epimastigotes to metacyclic trypomastigotes was quantified by staining with DAPI to distinguish the position of the kinetoplast by fluorescence microscopy. (H and I) Effect of *TcMCU* knockout on trypomastigote infection of Vero cells. There were no significant differences in percentages of infected Vero cells (H) or in the number of intracellular parasites per 100 host cells (I). (J) Effect of *TcMCU* knockout in amastigote replication after 48 h. Differences were not significant. Values in panels A to D and F to J are means ± SD (*n* = 3). *, *P* < 0.05; **, *P* < 0.01; ***, *P* < 0.001; n.s., not significant.

We initially evaluated the growth rate of epimastigotes in liver infusion tryptose (LIT) medium. *TcMCU-*KO cells grew at a slightly lower initial rate than wild-type cells or cells transfected with a scrambled sgRNA (Fig. 3A). We then tested their growth in low-glucose LIT medium. In this case, the initial growth rate of *TcMCU-*KO cells was significantly lower. Wild-type epimastigotes reached the stationary phase earlier (day 8) and then started dying, while *TcMCU*-KO cells reached the stationary phase later (day 11) and continued at steady state for a longer period (Fig. 3B). The longer survival of *TcMCU-*KO cells in low-glucose medium suggested that these cells could have access to an energy reserve. Amino acids or fatty acids derived from triglycerides have been proposed as a possible source of energy of epimastigotes under low-glucose conditions (1). We therefore investigated the presence of lipid droplets in *TcMCU-*KO cells under these conditions. Lipid droplets were more abundant in *TcMCU-*KO cells grown in LIT medium or in low-glucose LIT medium than in wild-type cells at the beginning of the stationary phase, suggesting triglycerides as a potential energy reserve of these cells (Fig. S3A and B).

10.1128/mBio.00574-17.4 FIG S3  Lipid droplets in epimastigotes and PCR analysis of *TcMCU-*KO trypomastigotes. (A) Staining with Nile red (red) of lipid droplets in wild-type (WT) and *TcMCU-*KO epimastigotes cultured in LIT or low-glucose LIT medium. *T. cruzi* epimastigotes were incubated in PBS containing 1.5 μg of Nile red (Sigma)/ml for 30 min at 28°C. Fluorescence optical images were captured with excitation at 510 nm and emission at 580 nm. Nuclei and kinetoplasts were labeled with DAPI (blue). Bars, 10 µm. (B) Number of lipid droplets detected per cell in LIT or low-glucose medium. At least 200 cells from three experiments with 20 random fields/experiment were analyzed (means ± SD; *n* = 3; ***, *P* < 0.001, Student’s *t* test). (C) PCR used to confirm *TcMCU-*KO genotype in trypomastigotes isolated after host cell infection, as described in Materials and Methods. The intact locus generates a PCR product of 909 bp in wild-type cells that is absent in *TcMCU-*KO trypomastigotes. Download FIG S3, TIF file, 2.2 MB. Copyright © 2017 Chiurillo et al. 2017 Chiurillo et al. This content is distributed under the terms of the Creative Commons Attribution 4.0 International license.

In contrast to *TcMCU-*KO cells, *TcMCUb-*KO cells grew in LIT medium at a significantly lower rate than wild-type cells or cells transfected with a scrambled sgRNA (Fig. 3C).

### Oxidative stress in *TcMCU-*OE and *TcMCUb*-OE cells.

Overexpression of *TcMCU* or *TcMCUb* resulted in higher basal mitochondrial Ca^2+^ concentration, as measured by Rhod-2 fluorescence (Fig. 3D). Mitochondrial Ca^2+^ overload is known to generate oxidative stress. Accordingly, reactive oxygen species (ROS) generation was increased in digitonin-permeabilized *TcMCU*-OE or *TcMCUb*-OE epimastigotes compared with control cells (wild type [WT]) when in the presence of 0.5 mM Ca^2+^ (Fig. 3E and F). They reached levels comparable to those produced by addition of antimycin A_1_, a known complex III inhibitor of the electron transport chain (Fig. 3E and F).

### Differentiation and infectivity of mutant cells.

To induce the differentiation of mutant epimastigotes into infective metacyclic trypomastigotes (metacyclogenesis), we incubated these cells in triatome artificial urine (TAU) medium as described in Materials and Methods. *TcMCU-*KO cells were able to differentiate to metacyclic trypomastigotes in a higher proportion than wild-type or *TcMCU-*OE cells (Fig. 3G). The *TcMCU-*KO metacyclic trypomastigotes were able to infect host cells. After several cycles of infection to obtain a sufficient amount of culture-derived trypomastigotes, we conducted *in vitro* infection assays to determine the percentage of infected cells after incubation with wild-type or *TcMCU-*KO trypomastigotes. Figures 3H and I show that there was no significant difference between infections with wild-type and *TcMCU-*KO trypomastigotes, and Fig. 3J shows that replication of amastigotes was not significantly affected by *TcMCU* knockout, demonstrating that *TcMCU* is not essential for differentiation, infectivity, or intracellular replication. PCR analysis of the *TcMCU*-KO trypomastigotes obtained from these tissue cultures confirmed the absence of *TcMCU* in these cells (Fig. S3C). In contrast, induction of metacyclogenesis in *TcMCUb-*OE cells was similar to that of wild-type cells but greatly diminished in *TcMCUb-*KO cells (Fig. 3G). Several attempts (*n* = 4) to infect tissue culture cells with *TcMCUb*-KO differentiated cells failed to recover significant amounts of trypomastigotes, suggesting that *TcMCUb* is important for infectivity.

### Complementation of the KO mutants.

**FIG 4:**
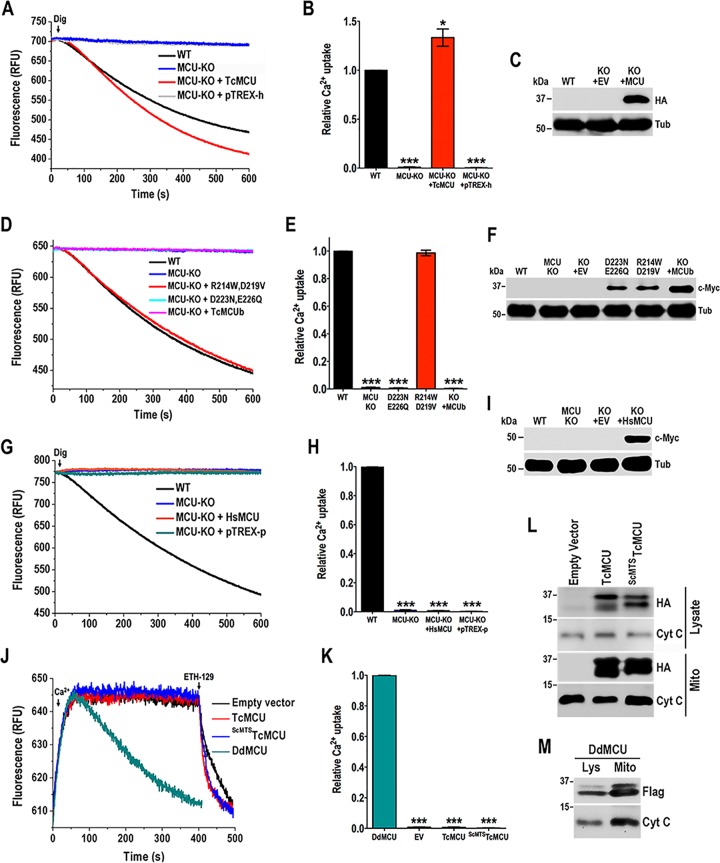
Complementation of the endogenous *TcMCU* and reconstitution of Ca^2+^ transport in yeast. (A) Ca^2+^ uptake reconstitution in digitonin-permeabilized *TcMCU-*KO epimastigotes transfected with pTREX-h and pTREX-h-*TcMCU_-PAM_-HA* (TcMCU). Experimental conditions were as in Fig. 1I. (B) Quantification of data in panel A. Relative Ca^2+^ uptake at 600 s compared with WT. (C) Western blot analysis of total protein extracts of wild-type (WT), *TcMCU-*KO plus pTREX-h empty vector (KO + EV), and *TcMCU-*KO plus *TcMCU_-PAM_-HA* (KO + MCU) epimastigotes, using anti-HA antibodies. Anti-α-tubulin antibodies were used as a loading control. (D) Ca^2+^ uptake reconstitution in digitonin-permeabilized *TcMCU-*KO epimastigotes transfected with *TcMCU*^R214W,D219V^ (MCU-KO + R214W,D219V), *TcMCU*^D223N,E226Q^ (MCU-KO + D223N,E226Q), or *TcMCUb* (MCU-KO + TcMCUb). Experimental conditions were as in Fig. 1I. (E) Quantification of data in panel D. Relative Ca^2+^ uptake at 600 s compared with WT (*TcMCU*^R214W,D219V^ [shown as R214W,D219V] and *TcMCU*^D223N,E226Q^ [shown as D223N,E226Q]). (F) Western blot analysis of total protein extracts of wild-type (WT), *TcMCU-*KO (MCU-KO), *TcMCU-*KO plus pTREX-h empty vector (KO + EV), *TcMCU*^D223N,E226Q^ (D223N,E226Q), *TcMCU*^R214W,D219V^ (R214W,D219V), and *TcMCUb* (KO + MCUb) epimastigotes, using anti-c-Myc antibodies. Anti-α-tubulin antibodies were used as a loading control. (G) Ca^2+^ uptake reconstitution in digitonin-permeabilized *TcMCU-*KO epimastigotes transfected with *HsMCU* or pTREX-p. Experimental conditions were as in Fig. 1I. (H) Quantification of data in panel G. Relative Ca^2+^ uptake at 600 s compared with WT. (I) Western blot analysis of total protein extracts of WT, *TcMCU-*KO (MCU-KO), *TcMCU-*KO plus pTREX-p empty vector (KO + EV), and *TcMCU*-KO plus *HsMCU* (KO + HsMCU) epimastigotes, using anti-c-Myc antibodies. Anti-α-tubulin antibodies were used as a loading control. (J) Ca^2+^ uptake by spheroplasts from yeast transformed with pACT2 empty vector, *TcMCU*, *^ScMTS^TcMCU*, or *DdMCU*. Experimental conditions were as described in Materials and Methods. (K) Quantification of data in panel J. Relative Ca^2+^ uptake at 350 s compared with DdMCU. (L) Western blot analysis of lysates and mitochondrial fractions of yeast complemented with empty vector, *TcMCU-HA* (TcMCU), or *^ScMTS^TcMCU-HA* (^ScMTS^TcMCU) using anti-HA antibodies. Anti-cytochrome *c* antibodies were used as a loading control. (M) Western blot analysis of lysates and mitochondrial fractions of yeast complemented with *DdMCU-*Flag (DdMCU) using anti-Flag antibodies. Anti-cytochrome *c* antibodies were used as a loading control. Values in panels B, E, H, and K are means ± SD (*n* = 3). *, *P* < 0.05; ***, *P* < 0.001.

To exclude the possibility that the *TcMCU* silencing phenotype is due to off-target effects, we investigated whether an exogenous *TcMCU* gene could complement the ablation of endogenous *TcMCU*. First, we demonstrated that the exogenous *TcMCU* (with changes in the PAM sequence to prevent disruption by CRISPR/Cas9), bearing a hemagglutinin (HA) epitope tag (*TcMCU_-PAM_-HA*), was targeted to the epimastigote mitochondrion. Immunofluorescence assays in Fig. S4A show that exogenous TcMCU-HA colocalizes with MitoTracker in the mitochondria. Then, we observed that mitochondrial Ca^2+^ transport in permeabilized *TcMCU-*KO epimastigotes expressing exogenous *TcMCU-HA* was significantly higher than that of wild-type cells (Fig. 4A and B). The expression of TcMCU-HA was confirmed by Western blot analysis (Fig. 4C). Then, we introduced mutations in highly conserved residues to evaluate their ability to restore Ca^2+^ transport in *TcMCU-*KO cells (Fig. S4B). Complementation with a *TcMCU*^R214W,D219V^ mutant, but not with the *TcMCU*^D223N,E226Q^ mutant of the DIME region of TcMCU, was able to restore mitochondrial Ca^2+^ uptake (Fig. 4D and E), indicating the importance of Asp^223^ and Glu^226^ of the DIME domain for Ca^2+^ transport. *TcMCU-*KO epimastigotes overexpressing *TcMCUb* failed to rescue Ca^2+^ uptake, suggesting that TcMCUb cannot replace TcMCU. Western blot analysis confirmed the expression of these proteins (Fig. 4F). We also performed the complementation of *TcMCU-*KO cells with human *MCU* (*Homo sapiens* MCU [HsMCU]), and we found that it was not effective in restoring mitochondrial Ca^2+^ transport (Fig. 4G and H), although the protein colocalized with MitoTracker in the mitochondria of epimastigotes (Fig. S4C) and Western blot analysis confirmed its expression (Fig. 4I).

10.1128/mBio.00574-17.5 FIG S4  Mitochondrial localization of TcMCU-HA and HsMCU-3×c-Myc in epimastigotes. (A) Fluorescence microscopy of exogenous TcMCU-HA expressed in *TcMCU-*KO epimastigotes using antibodies against HA (green) and MitoTracker as a mitochondrial marker (red). Nuclei and kinetoplast were labeled with DAPI (blue). Colocalization is shown in yellow (merge). (B) Schematic representation of MCU subunit domains. Sequence alignment of the DIME motif and flanking residues from MCU and MCUb. Numbers of critical conserved and amino acid substitutions are shown. TM, transmembrane domain; DIME, functional DIME motif. (C) Colocalization of HsMCU-3×c-Myc with MitoTracker using antibodies to c-Myc. Bars in panels A and C, 10 µm. Download FIG S4, TIF file, 1.6 MB. Copyright © 2017 Chiurillo et al. 2017 Chiurillo et al. This content is distributed under the terms of the Creative Commons Attribution 4.0 International license.

### Reconstitution of the mitochondrial calcium uniporter in *Saccharomyces cerevisiae*.

*Saccharomyces cerevisiae* does not possess an MCU, which makes it an ideal *in vivo* reconstitution system for studying the uniporter in a physiologically relevant organellar membrane (5, 19). Because the minimal components sufficient for *in vivo* MCU activity in trypanosomatids are unknown, we transformed *S. cerevisiae* with *TcMCU* to assess whether this protein is able to reconstitute yeast mitochondrial calcium uniporter activity on its own. We transformed yeast with MCU from *Dictyostelium discoideum* (*DdMCU*) to use as a positive control, as this protein was reported to be sufficient alone to reconstitute mitochondrial calcium uniporter activity in yeast (19). To ensure expression of *TcMCU* in yeast mitochondria, we created a chimeric gene, consisting of the mitochondrial targeting sequence (MTS) of yeast cytochrome oxidase 4 (ScCox4p) fused to *TcMCU* lacking its predicted MTS (*^ScMTS^TcMCU*) to obtain the expression of *TcMCU* in the yeast mitochondrial inner membrane. Extramitochondrial calcium levels were monitored with calcium green-5N, and we observed that mitochondria of yeast spheroplasts were able to take up Ca^2+^ in the presence of mitochondrial substrates (5 mM succinate, 5 mM malate, 5 mM pyruvate, 5 mM α-ketoglutarate, and 1 mM glutamate) only when complemented with *DdMCU* (Fig. 4J and K). However, neither *TcMCU*- nor ^*ScMTS*^*TcMCU*-transformed cells were able to take up Ca^2+^ under the experimental conditions tested, although the cells were able to take up Ca^2+^ after addition of the membrane-potential-dependent calcium ionophore ETH-129 (19) (Fig. 4J). The enrichment of these proteins in the mitochondrial fraction was confirmed by Western blot analysis (Fig. 4L and M). Our results suggest that *TcMCU* alone is unable to reconstitute mitochondrial Ca^2+^ uptake in yeast.

### Respiration of *TcMCU-*KO and *TcMCUb-*KO mutants.

**FIG 5:**
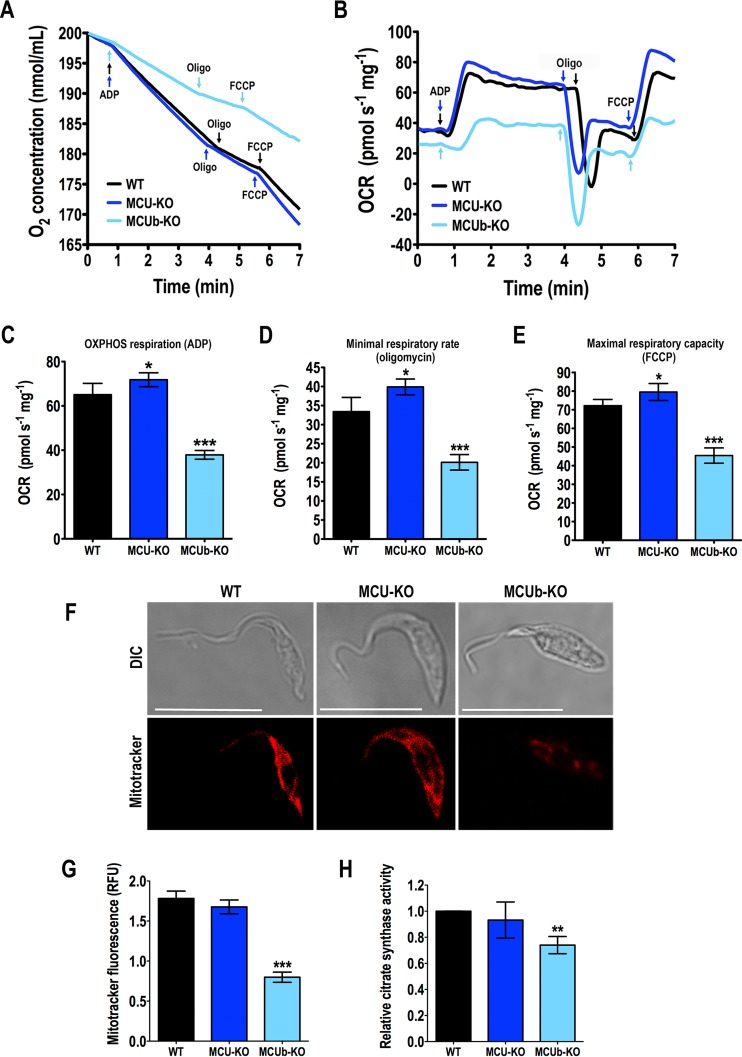
Mitochondrial changes in mutant epimastigotes. (A and B) Representative traces of oxygen uptake (A) and oxygen consumption rate (OCR) (B) by digitonin-permeabilized wild-type (WT), *TcMCU-*KO (MCU-KO), and *TcMCUb-*KO (MCUb-KO) epimastigotes. Additions were 100 μM ADP, 1 µg/ml oligomycin (Oligo), and 1.0 µM FCCP. (C to E) OCR after addition of ADP (respiration stimulated by oxidative phosphorylation [OXPHOS]), oligomycin (minimal respiratory rate), and FCCP (maximal respiratory capacity), respectively (*n* = 4), using the test system as in panels A and B. (F) Fluorescence microscopy of representative wild-type (WT), *TcMCU-*KO (MCU-KO), and *TcMCUb-*KO (MCUb-KO) epimastigotes labeled with MitoTracker Deep Red FM for estimation of mitochondrial mass. (G) Quantification of data in panel F. Relative fluorescence of MitoTracker Deep Red FM-labeled cells. Over 200 cells from three experiments with 20 random fields/experiment were analyzed (*n* = 3). (H) Relative citrate synthase activity of wild-type and mutant parasites, measured for enzymatic estimation of mitochondrial mass, considering *V*_max_ values of WT to be 1.0 (*n* = 5). Values in panels C to E, G, and H are means ± SD. *, *P* < 0.05; **, *P* < 0.01; ***, *P* < 0.001.

We measured oxygen uptake (Fig. 5A) and oxygen consumption rate (OCR) (Fig. 5B) under basal (state 2), ADP-stimulated (state 3), oligomycin-inhibited (state 4), and FCCP-stimulated (state 3u) conditions in wild-type and mutant digitonin-permeabilized cells in the presence of succinate. Wild-type and mutant mitochondria showed well-coupled respiration, although OCRs in the presence of ADP, oligomycin, and FCCP were significantly higher in *TcMCU-*KO and significantly lower in *TcMCUb-*KO mitochondria (Fig. 5C to E). Respiratory control rates (state 3/state 4) were 1.93 ± 0.05, 1.79 ± 0.09, and 1.92 ± 0.07 for WT, *TcMCU-*KO, and *TcMCUb-*KO cells, respectively (*n* = 4). The lower OCR of *TcMCUb-*KO mitochondria correlated with lower staining of the mitochondria with MitoTracker Deep Red FM, a dye currently used to estimate mitochondrial mass (20) (Fig. 5F and G), as well as with lower activity of citrate synthase, another mitochondrial mass indicator (21) (Fig. 5H), suggesting a lower mitochondrial mass in these cells.

### Autophagy in *TcMCUb-*KO cells.

**FIG 6:**
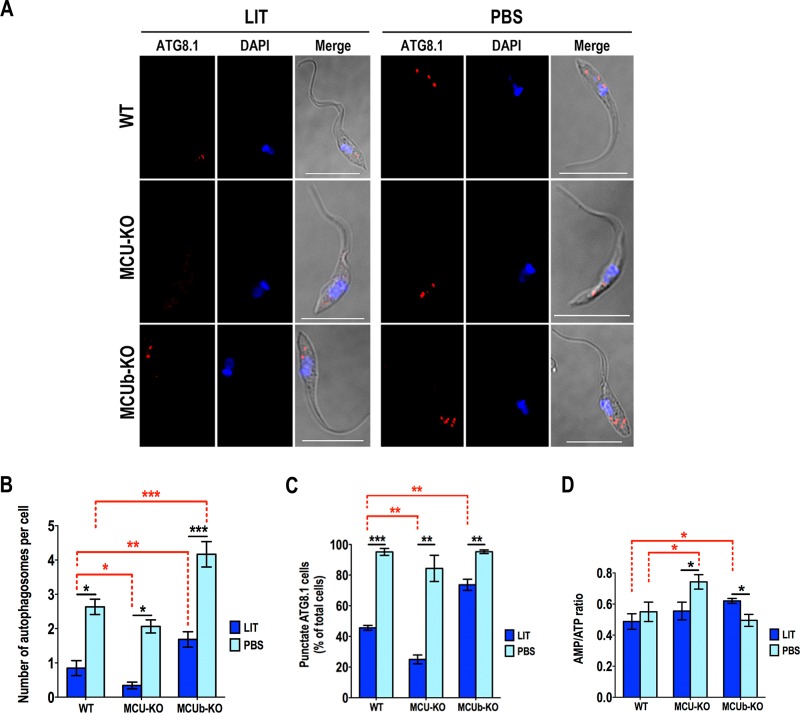
Autophagy changes in mutant epimastigotes. (A) Representative fluorescence microscopy images of wild-type (WT), *TcMCU-*KO (MCU-KO), and *TcMCUb-*KO (MCUb-KO) epimastigotes labeled with anti-TcATG8.1 antibody (red) after incubation in LIT medium or PBS for 16 h. DAPI staining (blue) and merged images on a differential interference contrast (DIC) background (Merge) are also shown. Bars, 10 µm. (B) Number of autophagosomes per cell under different conditions shown in panel A. (C) Percentage of cells with autophagosomes under conditions shown in panel A. (D) Comparison of AMP/ATP ratios between WT, MCU-KO, and MCUb-KO epimastigotes incubated in LIT medium or PBS for 16 h. For panels B to D, over 200 cells from wild-type (WT), *TcMCU-*KO (MCU-KO), and *TcMCUb-*KO (MCUb-KO) epimastigotes from three experiments with 20 random fields/experiment were analyzed. Values in panels B to D are means ± SD (*n* = 3). *, *P* < 0.05; **, *P* < 0.01; ***, *P* < 0.001.

Autophagy has been reported as a starvation response in different trypanosomatids (22, 23). In addition, block of mitochondrial Ca^2+^ uptake was shown to increase autophagy as a survival mechanism (24, 25). Huang et al. (10) recently showed that knockdown of *MCU* in *T. brucei* procyclic forms increased the expression of autophagy markers under starvation condition. We evaluated autophagy in these cells by immunofluorescence microscopy (Fig. 6A) using antibodies against the autophagy marker Atg8.1, which is the ortholog of LC3-II in mammalian cells (26). Then, we quantified the number of autophagosomes per cell (Fig. 6B) and the number of cells containing autophagosomes (Fig. 6C). *TcMCUb-*KO cells had significantly increased numbers of autophagosomes per cell when incubated under starvation conditions, while lower levels were found in *TcMCU-*KO cells than in wild-type cells (Fig. 6A to C). Moreover, *TcMCUb*-KO cells showed an increased number of autophagosomes/cell and a higher percentage of cells with autophagosomes even when cultured in rich medium. It has been proposed that in vertebrate cells a lower mitochondrial Ca^2+^ transport capacity leads to an increase of the AMP/ATP ratio and stimulation of the AMP-dependent protein kinase (AMPK) signaling axis that stimulates autophagy (24). However, knockout of both *TcMCU* and *TcMCUb* abolished mitochondrial Ca^2+^ transport while only *TcMCUb-*KO cells showed increased autophagy. In agreement with these results, there was no correlation between the AMP/ATP ratio of these cells (Fig. 6D) and autophagy (Fig. 6A to C), ruling out this potential mechanism of autophagy stimulation in *T. cruzi*.

## DISCUSSION

Our studies have shown that the mitochondrial calcium uniporter (TcMCU) is not essential for *T. cruzi* epimastigote growth in rich medium, for differentiation (metacyclogenesis) under well-established starvation conditions, and for trypomastigote host cell infection and intracellular replication, although it is needed for optimal exponential growth of epimastigotes. No mitochondrial Ca^2+^ transport was detected in *TcMCU-*KO epimastigotes, while the mitochondrial membrane potential (ΔΨ_m_) was unaffected in these cells. In contrast to these results, TcMCUb was required for optimal growth in rich medium, for metacyclogenesis under starvation conditions, and for trypomastigote host cell infection. However, as occurs with *TcMCU-*KO cells, mitochondria of *TcMCUb-*KO epimastigotes were unable to transport Ca^2+^ while their ΔΨ_m_ was not altered. Additional differences between these KO cells were also found. *TcMCUb-*KO cells had lower respiratory activity, a higher AMP/ATP ratio in rich medium, reduced mitochondrial mass, and increased autophagy, suggesting that *MCUb* has additional bioenergetic roles independent of Ca^2+^ transport. Overexpression of *TcMCUb* or *TcMCU* resulted in higher rates of Ca^2+^ transport, no effects on ΔΨ_m_, and no dominant negative effect of TcMCUb on mitochondrial Ca^2+^ transport, in contrast to previous results described for HeLa cells (13). The two proteins interact, as shown by their coimmunoprecipitation.

MICU1 has been shown to have a gatekeeping effect on MCU (27, 28), and the increased matrix Ca^2+^ observed after overexpression of *TcMCU* could be the result of a change in the stoichiometry of MCU/MICU1. A similar situation could be occurring in *TcMCUb*-overexpressing cells.

The lack of essentiality of MCU in epimastigotes grown in rich medium and their increased survival in stationary phase under low-glucose conditions is interesting. It has been previously reported that epimastigotes use glucose from the medium during their exponential phase of growth and that once glucose is depleted and cells enter the stationary phase, they use tricarboxylic acid (TCA) cycle intermediates (29). All the main stages of *T. cruzi* have a high endogenous rate of respiration, suggesting that the organisms contain an energy reserve. Early reports ruled out the presence of a carbohydrate polymer (1, 30). Oliveira et al. (31) first suggested the possibility that triglycerides could be the energy reserve, which would be similar to the situation in another stercorarian trypanosome, *Trypanosoma lewisi*, which also has a high endogenous rate of respiration and high triglyceride content (32). However, we found that lipid droplets increase, not decrease, in *TcMCU-*KO cells. As the presence of lipid droplets was evaluated at the beginning of the stationary phase, one possibility is that they could allow *TcMCU*-KO cells to survive longer because they were not consumed yet at that point. On the other hand, it is known that epimastigotes have a large pool of free amino acids that they use to maintain their osmolarity (33) and that protein degradation occurs under starvation conditions (1). Increased amino acid oxidation through the activity of the TCA cycle would bypass the need for the Ca^2+^-regulated formation of acetyl coenzyme A (CoA) from pyruvate (10). The higher ATP production via this pathway would explain the better survival of the cells when grown in low-glucose medium. The intracellular amastigotes appear to use fatty acids as an energy source, taking into account the increased levels of fatty acid oxidation enzymes in these stages (34). Amastigotes could then also bypass the need for the Ca^2+^-regulated pyruvate dehydrogenase to obtain energy through the TCA cycle. The predominance of fatty acid oxidation over glycolysis in the intracellular stages would explain the nonessential role of *TcMCU* for infection and replication.

Metacyclogenesis is usually stimulated under glucose-depleted conditions, and we detected a higher rate in *TcMCU-*KO cells. This higher rate of differentiation could be related to the lower ability of these cells to use endogenous substrates in low-glucose medium in contrast to *TcMCU-*OE cells. The increased metacyclogenesis observed in *TcMCU*-KO cells could also explain its longer survival during the stationary phase in low-glucose medium. On the other hand, the reduced metacyclogenesis of *TcMCUb-*KO cells correlated well with their inefficient ability to invade host cells.

To our knowledge, we report for the first time the complementation of a gene knockout (*TcMCU-*KO) obtained by CRISPR/Cas9 in *T. cruzi*. Complementation of *TcMCU-*KO cells with an exogenous copy of *TcMCU* but not with the gene mutated in the nucleotides encoding amino acids Asp^223^ and Glu^226^ of the DIME domain was able to restore mitochondrial Ca^2+^ transport, confirming that as demonstrated in mammals, these two conserved acidic residues of the DIME motif are also key for TcMCU-mediated Ca^2+^ uptake (8, 13). However, a mutant including a substitution at the acidic residue Asp^219^ (*TcMCU*^R214W/D219V^; Glu^257^ in HsMCU), which is highly conserved within MCU orthologs and has been proposed as a critical residue for Ca^2+^ transport (8, 13), rescued Ca^2+^ transport in *TcMCU-*KO epimastigotes. Similarly, a recent report showed that the *HsMCU*^E257A^ mutant was able to rescue Ca^2+^ transport in HEK-293T MCU-KO cells, which is consistent with the predicted nuclear magnetic resonance (NMR)/electron microscopy (EM) structure of the MCU that exposes this residue outside the channel entrance (35).

Moreover, human *MCU* was unable to restore mitochondrial Ca^2+^ transport in *TcMCU-*KO cells. *TcMCU* alone, in contrast to *DdMCU* (19), could not reconstitute mitochondrial Ca^2+^ transport in yeast mitochondria. As *T. cruzi* lacks orthologs of EMRE (36) or MCUR1 (37, 38), this result suggests that, as in the case of human MCU (19), other, still-unknown subunits of the complex might be necessary for full reconstitution of mitochondrial Ca^2+^ transport in yeast by *TcMCU*.

An increase in autophagy was observed in *TcMCUb-*KO but not in *TcMCU-*KO cells when incubated under either rich or starvation conditions. In vertebrate cells, the lack of MCU-mediated Ca^2+^ uptake promotes autophagy (37, 38). An increase in the AMP/ATP ratio produced by downregulation of the TCA cycle by inhibition of the MCU could stimulate the phosphorylation of the AMP-dependent kinase (AMPK) and promote autophagy (24). However, we did not find such a correlation in *T. cruzi*, as *TcMCU-*KO cells have higher AMP/ATP ratios under starvation conditions and no difference in autophagy while *TcMCUb-*KO cells do not have differences in AMP/ATP ratios but exhibit higher autophagy than wild-type cells. These results suggest that this pathway might not be operative in *T. cruzi* and are in agreement with a reported AMPK-independent autophagy pathway reported recently in *T. brucei* (39).

It is possible that phenotypic changes in the *TcMCUb-*KO mutants may have been caused indirectly by adaptation to the loss of the gene that were not seen in the *TcMCU*-KO mutants because of the occurrence of more beneficial compensatory mutations in these cells. However, our attempts to complement *TcMCUb*-KO mutants with an exogenous *TcMCUb* gene devoid of the PAM motif did not result in selected parasites, probably due to the fragility of these cells and their inability to resist the electroporation stress (data not shown).

The mitochondria of *TbMCU-*OE epimastigotes have an increased ability for Ca^2+^ uptake, which results in Ca^2+^ overload and oxidative stress, and similar results were observed in *TcMCUb*-OE cells. These results agree with those obtained in HeLa cells (7) and *T. brucei* (10). Mitochondrial Ca^2+^ overload has also been proposed as the link between complement deposition and triggering of cell death in *T. cruzi* epimastigotes (40). Accumulation of Ca^2+^ in the mitochondrion leads to a decrease in cell respiration, dissipation of the inner membrane potential, and increased reactive oxygen species production (40).

In contrast with the results obtained in HeLa cells (13), where overexpression of *MCUb* has a dominant negative effect on mitochondrial Ca^2+^ transport, overexpression of *TcMCUb* led to increased Ca^2+^ uptake without affecting the mitochondrial membrane potential. Knockout of *TcMCUb* abolished mitochondrial Ca^2+^ transport, and the cells had a lower growth rate than control cells. Moreover, as mentioned above, we introduced in TcMCU amino acid substitutions (R214W/D219V) that are conserved in its paralog TcMCUb and considered critical to determine the dominant negative effect of the mammalian protein (13). However, we found that *TcMCU*^R214W/D219V^, but not *TcMCUb*, was able to restore mitochondrial Ca^2+^ transport in *TcMCU-*KO cells, suggesting that these substitutions are not enough to abolish Ca^2+^ transport.

In summary, our results indicate that *TcMCU* is not essential for growth, differentiation, infectivity, and intracellular replication of *T. cruzi*, although it could be important under stress situations, such as for growth in low-glucose medium. This could be relevant for epimastigotes grown in the insect’s gut, in which glucose is supposed to be scarce (41). In contrast to what has been described in some mammalian cells, where MCUb acts as a dominant negative subunit of the MCU complex (13), mitochondrial Ca^2+^ transport is enhanced in *TcMCUb-*OE epimastigotes and abolished in *TcMCUb-*KO cells. *TcMCUb*-KO cells have additional phenotypic alterations that suggest other bioenergetic roles besides mitochondrial Ca^2+^ transport.

## MATERIALS AND METHODS

### Culture methods.

*T. cruzi* epimastigotes (Y strain) were grown at 28°C in liver infusion tryptose (LIT) medium (42) (5.4 mM KCl, 150 mM NaCl, 24 mM glucose, 5% [vol/vol] liver extract, 0.02% [wt/vol] hemin, 2% [wt/vol] yeast extract, 1.5% [wt/vol] tryptose), supplemented with 10% heat-inactivated fetal bovine serum (FBS). To obtain a low-glucose LIT medium, glucose was not added. We determined the growth rate of *T. cruzi* epimastigotes by counting cells in a Neubauer chamber with a starting culture of 1 × 10^6^ epimastigotes. Tissue culture cell-derived trypomastigotes were obtained from Vero cells infected with metacyclic trypomastigotes obtained as described below. *T. cruzi* trypomastigote forms were collected from the culture medium of infected host cells, using a modification of the method of Schmatz and Murray (43) as described previously (44). Vero cells were grown in RPMI supplemented with 10% fetal bovine serum and maintained at 37°C with 5% CO_2_.

### Metacyclogenesis.

We followed the protocol described by Bourguignon et al. (45) with minor modifications. Epimastigotes were obtained after 4 days in LIT medium and submitted to a stress (incubation for 2 h in a medium containing 190 mM NaCl, 17 mM KCl, 2 mM MgCl_2_, 2 mM CaCl_2_, 0.035% sodium bicarbonate, 8 mM phosphate, pH 6.9, at room temperature; triatome artificial urine [TAU] medium). After this stress, parasites were incubated for 96 h in TAU 3AAG medium (which consists of the above-described TAU medium supplemented with 10 mM l-proline, 50 mM sodium l-glutamate, 2 mM sodium l-aspartate, and 10 mM glucose). To increase the number of metacyclic forms to infect Vero cells, the contents of the flask were collected and resuspended in medium containing fresh fetal bovine serum and incubated at 37°C for 20 h. The complement in the FBS kills epimastigotes while metacyclic trypomastigotes survive. Samples were harvested from the TAU 3AAG plus FBS-containing medium at days 5 and 10 of cultivation.

### *In vitro* infection assay.

Gamma-irradiated (2,000-rad) Vero cells (4.5 × 10^5^ cells) were plated onto sterile coverslips in a 12-well plate and incubated overnight at 35°C, 7% CO_2_, in RPMI medium plus 10% fresh fetal bovine serum. Tissue culture-derived trypomastigote collections were incubated at 4°C overnight to allow amastigotes to settle from swimming trypomastigotes. Trypomastigotes from the supernatants of these collections were counted and used to infect the coverslips at a ratio of 50 parasites to 1 host cell. At 4 h postinfection, coverslips were washed extensively with Dulbecco’s Hanks’ solution, followed by phosphate-buffered saline (PBS), pH 7.4, to remove any extracellular parasites. Coverslips were fixed immediately in 4% paraformaldehyde in PBS, pH 7.4, at 4°C for 30 min. Coverslips were washed once with PBS and mounted onto glass slides in Fluoromount G containing 15 µg/ml of 2-(4-aminophenyl)-1H-indole-6-carboxamidine (DAPI), which stains host and parasite DNA. Coverslips were viewed on an Olympus BX60 microscope to quantify the number of host cells that contained intracellular parasites and the number of intracellular parasites per cell in randomly selected fields. Three hundred host cells were counted per sample in three independent experiments. To quantify amastigote replication, the following modifications were used: host cells were infected at a ratio of 10 parasites to 1 host cell, and coverslips were allowed to incubate for 48 h postinfection at 35°C, 7% CO_2_, prior to fixation and DAPI staining.

### *TcMCU*-KO.

Chimera single guide RNA (sgRNA) sequences to target the *TcMCU* gene (TryTripDB identifier [ID] TcCLB.503893.120) were PCR amplified from plasmid pUC_sgRNA, containing the sgRNA backbone sequence (82 bp) (46). One specific protospacer was included in the forward primer (primer 1 [see Table S1 in the supplemental material]) while using a common reverse primer (primer 2 [Table S1]) for sgRNA amplification. These primers also contained a BamHI restriction site for cloning into Cas9/pTREX-n (14) upstream of the HX1 transsplicing signal (47) to generate the *TcMCU*-sgRNA/Cas9/pTREX-n construct. The sgRNA orientation was verified by PCR using the specific TcMCU-sgRNA forward primer and the HX1 reverse primer (14), which annealed on the HX1 region of the vector (primer 3 [Table S1]). Positive clones that generate a 190-bp PCR fragment were also sequenced. A scrambled sgRNA (Sc-sgRNA/Cas9/pTREX-n), obtained with primers 2 and 4 (Table S1), was used as a control. The Cas9/pTREX-n vector contains the *Streptococcus pyogenes* Cas9 sequence with a twice-repeated simian virus 40 (SV40) nuclear localization signal (2×NLS) and green fluorescent protein (GFP) (14), thus generating mutant cell lines with green fluorescent nuclei after transfection. The molecular construct (DNA donor) to promote homologous directed repair and ablation of the target gene after the double-strand break (DSB) induced by Cas9 was obtained using a recombinant PCR strategy. Briefly, the 5′ and 3′ UTRs of the *TcMCU* gene (490 and 529 bp, respectively) were amplified by PCR using *T. cruzi* genomic DNA as the template (primers 7 to 10 [Table S1]). The open reading frame (ORF) of the blasticidin *S*-deaminase gene (*Bsd*) was also amplified by PCR from pTREX-b vector as the template using primers containing 20 nucleotides (nt) overlapping with the 5′ and 3′ flanking sequences of the *TcMCU* gene (primers 5 and 6 [Table S1]). The three PCR fragments (5′-*TcMCU* flanking sequence, blasticidin-resistant gene, and 3′-*TcMCU* flanking sequence) were linked together by sequential PCR using primers 7 and 10 (Table S1). The final PCR product was cloned into pGEM-T Easy vector (Promega). After sequencing verification of positive clones, circular constructs *TcMCU*-sgRNA/Cas9/pTREX-n and the *Bsd* cassette in pGEM-T Easy were used to cotransfect *T. cruzi* epimastigotes. After 5 weeks of selection with 250 µg/ml G418 and 10 µg/ml blasticidin, *TcMCU* gene ablation was verified by PCR using primers 11 and 12 (Table S1). Alternatively, to confirm *TcMCU* gene KO in trypomastigotes obtained from infected cells, we amplified the entire *TcMCU* ORF (909 bp) by PCR (primers 15 and 19 [Table S1]).

### *TcMCUb*-KO.

An sgRNA to target the sequence coding for the hypothetical TcMCUb protein (TryTripDB ID TcCLB.416883.9) was amplified by PCR (primers 31 and 2 [Table S1]). Following the same strategy mentioned above for *TcMCU*, we obtained the *TcMCUb*-sgRNA/Cas9/pTREX-n construct. The DNA donor cassette designed to promote homologous directed repair and disruption of *TcMCUb* was obtained by PCR using a set of long primers (ultramers) containing 120 nucleotides, from which 100 nucleotides correspond to the region from nt −20 to +80 (forward ultramer) and nt +510 to +610 (reverse ultramer) of the *TcMCUb* ORF. Each one of the ultramers had 20 nt annealing on the *Bsd* gene (primers 32 and 33 [Table S1]). Gene disruption of *TcMCUb* was verified by PCR using primers 34 and 35 (Table S1).

### Generation of *TcMCU/TcMCUb*-overexpressing cell lines in *T. cruzi*.

For gain-of-function analysis of *TcMCU/TcMCUb*, *T. cruzi* epimastigotes were transfected with the trypanosome expression vector pTREX-n (47, 48) containing the corresponding genes. The molecular construct for *TcMCU* overexpression was made as follows: the full sequence of *TcMCU* was PCR amplified using primers 15 and 16 (Table S1) and *T. cruzi* gDNA as the template. The purified fragment of 909 bp was digested with EcoRI and HindIII restriction enzymes and cloned into the pTREX-n vector linearized with the same enzymes. The molecular construct for *TcMCUb* overexpression was made as follows: the full sequence of *TcMCUb* was PCR amplified using primers 38 and 39 (Table S1) and *T. cruzi* gDNA as the template. For this purpose, we used the genomic sequence (KB205522 from nucleotide 139,788 to 140,579, TryTripDB) from *T. cruzi* strain Esmeraldo to design the reverse primer, because the *MCUb* gene 3′ end and 3′ UTRs are missing in the TcCLB.416883.9 sequence. The purified fragment of 792 bp was digested with XbaI and SalI restriction enzymes to be cloned into a version of the pTREX-n vector that includes a 3×HA epitope tag coding sequence. The pTREX-n vector was linearized with XbaI and XhoI enzymes, and the *TcMCUb* gene was cloned in frame with the 3×HA sequence for C-terminal tagging, allowing the detection of the overexpressed protein with anti-HA antibody. For this purpose, we previously cloned the 3×HA tag sequence, excised from pMOTag-4H vector (49) with XhoI and SalI enzymes, into pTREX-n by using the XhoI restriction site.

### Molecular constructs to complement *TcMCU-*KO mutants.

To revert the phenotype exhibited by *TcMCU*-knockout epimastigotes, we obtained the *TcMCU_-PAM_-HA* construct to reconstitute the ablated *TcMCU* gene. Following a two-step-PCR strategy, we eliminated the PAM sequence (CGG to CCT) specific for the *TcMCU-*sgRNA used to obtain the *TcMCU*-KO cells, therefore avoiding constitutively expressed Cas9 to target the inserted sequence (Table S1, primers 15 and 17 to 19). The fragment obtained by overlap extension PCR was digested with EcoRI and HindIII and then cloned into pTREX-h (which confers hygromycin resistance) digested with the same enzymes. We also included a C-terminal HA tag in order to detect the overexpressed protein using anti-HA antibody.

Furthermore, we attempted to reconstitute the ablated *TcMCU* gene in *TcMCU*-KO mutants with the following. For *TcMCUb*, primers 38 and 39 (see Table S1 in the supplemental material) were used to amplify from *T. cruzi* gDNA the *TcMCUb* gene that was digested with XbaI and SalI enzymes and cloned in frame with the 3×c-Myc tag sequence into pTREX-h digested with XbaI and XhoI. For *TcMCU*^D223N,E226Q^ and *TcMCU*^R14W,D219N^, the generation of pTREX-h-*TcMCU*^D223N,E226Q^-3×c-Myc was done by overlap extension PCR using the TcMCU_-PAM_-HA plasmid as a template and primers 20 to 23 (Table S1). Following a similar strategy, the pTREX-h-*TcMCU*^R14W,D219N^-3×c-Myc was obtained using primers 20, 21, 24, and 25 (Table S1). For the human *MCU* gene, we purchased from GenScript (USA) a cDNA clone corresponding to the *Homo sapiens MCU* variant 1 (GenBank accession number NM_138357) of 1,100 bp. The *HsMCU* fragment amplified with primers 26 and 27 (Table S1) was digested and cloned into pTREX-p (which confers puromycin resistance) by EcoRI and XhoI restriction sites in frame with the 3×c-Myc tag sequence.

For this purpose, we first generated pTREX-h and pTREX-p vectors that were not available (Table S1, primers 40 to 48). As templates to amplify *GAPDH* UTRs and hygromycin and puromycin genes, we used pTREX-n, pMOTag-4H (49), and pMOTag-23M (49) plasmids, respectively. The 3×c-Myc tag was excised from the pMOTag-23M vector (49) with XhoI and SalI enzymes and subsequently cloned into pTREX-h and pTREX-p by the XhoI restriction site.

### Statistical analysis.

Values indicated in the figure legends are means ± standard deviations (SD) of *n* biological experiments (*n* is indicated in the figure legends), and the significance of their differences was evaluated by the Student *t* test.

10.1128/mBio.00574-17.1 TEXT S1 Supplemental methods. Download TEXT S1, DOCX file, 0.1 MB. Copyright © 2017 Chiurillo et al. 2017 Chiurillo et al. This content is distributed under the terms of the Creative Commons Attribution 4.0 International license.

10.1128/mBio.00574-17.6 FIG S5  Full blots shown in the article. Download FIG S5, TIF file, 2.6 MB. Copyright © 2017 Chiurillo et al. 2017 Chiurillo et al. This content is distributed under the terms of the Creative Commons Attribution 4.0 International license.
