# Supplementary material for: Different Roles of Mitochondrial Calcium Uniporter Complex Subunits in Growth and Infectivity of Trypanosoma cruzi
Source: mBio. 2017 May 9;8(3):e00574-17. doi: 10.1128/mBio.00574-17 (PMC5424207; doi:10.1128/mBio.00574-17)
Supplement: TABLE S1 [file mbo002173298st1.docx]

**Table S1.** Oligonucleotides used in this work.

|  | **Primer name** | **Sequence (5’ → 3’)** |
| --- | --- | --- |
| 1 | **Fw TcMCU_sgRNA** | GATC*GGATCC***ATGTTTTTGTAATACGGCCG**GTTTTAGAGCTAGAAATAGC |
| 2 | **Rv sgRNA** | CAGT*GGATCC*AAAAAAGCACCGACTCGGTG |
| 3 | **Rv_HX1-pTREX** | TAATTTCGCTTTCGTGCGTG |
| 4 | **Fw_Scramble_sgRNA** | GATC*GGATCC*GCACTACCAGAGCTAACTCAGTTTTAGAGCTAGAAATAGC |
| 5 | **Fw Bsd + TcMCU 5' UTR** | TAGAACAAAACGCTACGGATGAAATGGCCAAGCCTTTGTCTCAAG |
| 6 | **Rv Bsd + TcMCU 3' UTR** | CACGCCCAAAGTTTTTCCTTGCTTAGCCCTCCCACACATAACC |
| 7 | **Fw TcMCU 5' UTR** | CACTTTCAATCCGATGGGTAGAG |
| 8 | **Rv TcMCU 5' UTR** | ATTTCATCCGTAGCGTTTTGTTCTA |
| 9 | **Fw TcMCU 3' UTR** | GCAAGGAAAAACTTTGGGCGTG |
| 10 | **Rv TcMCU 3' UTR** | TAAACCGTTGTGGCGATTGG |
| 11 | **TcMCU_5' KO check** | AGGACCTCAAAGTTTGCTACCTCG |
| 12 | **TcMCU_3' KO check** | GGAAAGCAAACACATGGC |
| 13 | **Fw TcMCU probe** | CGATGGACAGTGCTGTTGG |
| 14 | **Rv TcMCU probe** | CGTCACTATTTTCTACATGGC |
| 15 | **Fw TcMCU_EcoRI** | TATA*GAATTC*ATGCGCGTAGGGCCATC |
| 16 | **Rv TcMCU_HindIII** | CTAG*AAGCTT*AGTGTTTTTTCATCCATTCCTTG |
| 17 | **Fw TcMCU_w/o PAM** | TTTGTAATACGGCCGC*ct*TGTTGTGCAAGAAGTCTC |
| 18 | **Rv TcMCU_w/o PAM** | ACTTCTTGCACAACA*ag*GCGGCCGTATTACAAAAAC |
| 19 | **Rv TcMCU_HA_HindIII** | CTAG*AAGCTT*ACGCGTAGTCCGGCACGTCGTACGGGTAGTGTTTTTTCATCCATTCCTTG |
| 20 | **Fw TcMCU_XbaI** | GATC*TCTAGA*TGCGCGTAGGGCCATCTATTCG |
| 21 | **Rv TcMCU_w/o STOP_XhoI** | GATC*CTCGAG*GTGTTTTTTCATCCATTCCTTG |
| 22 | **Fw TcMCU 223/226 mutant** | TTTGATTGG*a*ATACCATG*c*AGCCCGTATCGTAC |
| 23 | **Rv TcMCU 223/226 mutant** | ATACGGGCT*g*CATGGTAT*t*CCAATCAAAATCAAAG |
| 24 | **Fw MCU 214/219 mutant** | TATTTCG*t*G*g*CTGACGTTCTTTG*tg*TTTGATTGGGATACCATGGAG |
| 25 | **Rv MCU 214/219 mutant** | AATCAAA*ca*CAAAGAACGTCAG*c*C*a*CGAAATAATTGCCAGTTGCG |
| 26 | **Fw HsMCU_EcoRI** | TATA*GAATTC*ATGGCGGCCGCCGCAGGTAGAT |
| 27 | **Rv HsMCU_w/o STOP_XhoI** | CTAG*CTCGAG*ATCTTTTTCACCAATTTGTCGG |
| 28 | **Fw TcMCU_pACT2_HindIII** | AATCAACTCC*AAGCTT*ATGCGCGTAGGGCCATCTA |
| 29 | **Fw ScTcMCU_pACT2_HindIII** | AATCAACTCC*AAGCTT*ATGCTTTCACTACGTCAATCTATAAGATTTTTCAAGCCAGCCACAAGAACTTTGTGTAGCTCTAGATATCTGCTTCAGATTTCTTCTGCGGGGAAATG |
| 30 | **Rv TcMCU_pACTHA_HindIII** | AAGAAGTCCA*AAGCTT*CTATGCGTAATCGGGCACATCGTATGGGTAGTGTTTTTTCATCCATTCCTTG |
| 31 | **Fw TcMCUb_sgRNA** | GATC*GGATCC***GAACTTCCTACCCTTTCTCG**GTTTTAGAGCTAGAAATAGC |
| 32 | **Fw TcMCUb ultramer** | **TTGTCGGGTGGTGTGAAGCTCACGATTCAGAAGACACGAGCGCTATGGGACTCATCAATGAGTGTGCAATGTCTCAAAAGAAACTGCGGGCACGATGTG**ATGGCCAAGCCTTTGTCTCAAG |
| 33 | **Rv TcMCUb ultramer** | **GTGTACATGGTGAAAATATCTGATCATCAATGGCATGAAAGGTGCAATTTCTCCGGCACACAAGGGAATACGCGTAGACACAGAGGGCCGTGAATGTCGT**TTAGCCCTCCCACACATAACC |
| 34 | **Fw TcMCUb_disrup check** | ATGATTCAGAAGACACGAGC |
| 35 | **Rv TcMCUb_disrup check** | AGACATTTCCCTGGCGAC |
| 36 | **Fw TcMCUb probe** | TTGGATTGCAGCGGTAAAAGG |
| 37 | **RV TcMCUb probe** | ACAAAATAACAGACAGGCTCC |
| 38 | **Fw TcMCUb_XbaI** | GATC*TCTAGA*ATGATTCAGAAGACACGAGC |
| 39 | **Rw TcMCUb_w/o STOP_SalI** | GATC*GTCGAC*CTTCATTCCTTTGGTATTACTGG |
| 40 | **FW_G1_Hyg** | CACCGAGCGACCCTGCAGCCAATATGAAAAAGCCTGAACTCACCGCGAC |
| 41 | **FW_G1** | AAGCTTATCGATACCGTCGACCTC |
| 42 | **Fw_G2** | GGGGATCGATCCGGAACAACCTG |
| 43 | **Rv_G1** | CATATTGGCTGCAGGGTCGCTCGG |
| 44 | **RV_G2_NheI_SacI** | CTAG*GAGCTC*CCACACG*GCTAGC*ATACTC |
| 45 | **Rv_Hyg_G2** | GGTTGTTCCGGATCGATCCCCCTAGTCCTTTGCCCTCGGACGAGTG |
| 46 | **FW_G1_Pac** | CACCGAGCGACCCTGCAGCCAATATGACCGAGTACAAGCCCAC |
| 47 | **Rv_G1_Pac** | GTCATATTGGCTGCAGGGTCGCTCGG |
| 48 | **Rv_Pac_SpeI** | TCAG*ACTAGT*TCAGGCACCGGGCTTGCGGGTC |

Bold uppercase: specific protospacer; italic uppercase: restriction site; bold underlined uppercase: gene-specific homologous region; italic lower case: mismatch nucleotides
