# Supplementary figures and images for: Different Roles of Mitochondrial Calcium Uniporter Complex Subunits in Growth and Infectivity of Trypanosoma cruzi
Source: mBio. 2017 May 9;8(3):e00574-17. doi: 10.1128/mBio.00574-17 (PMC5424207; doi:10.1128/mBio.00574-17)

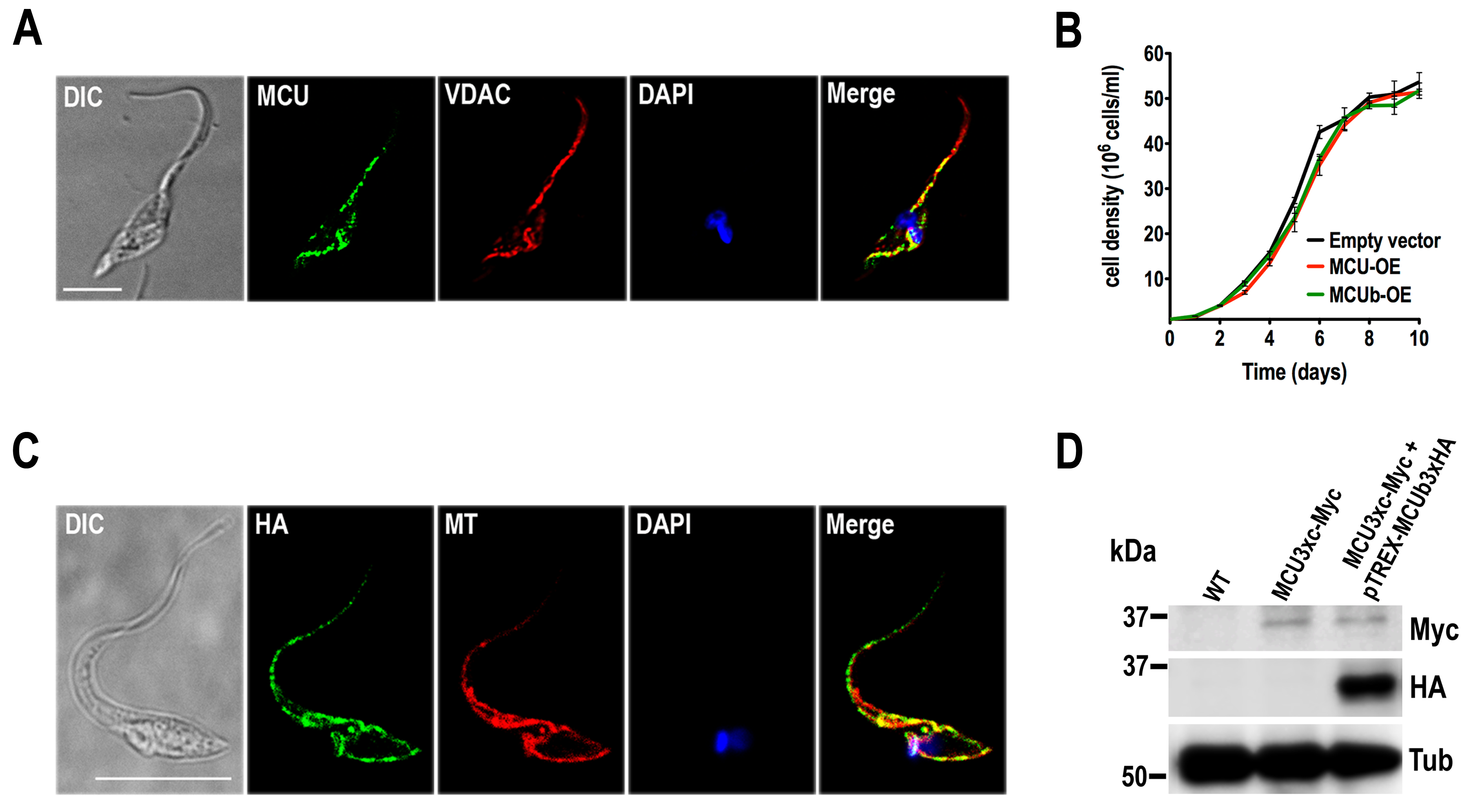

Supplement: FIG S1 [file mbo002173298sf1.tif]

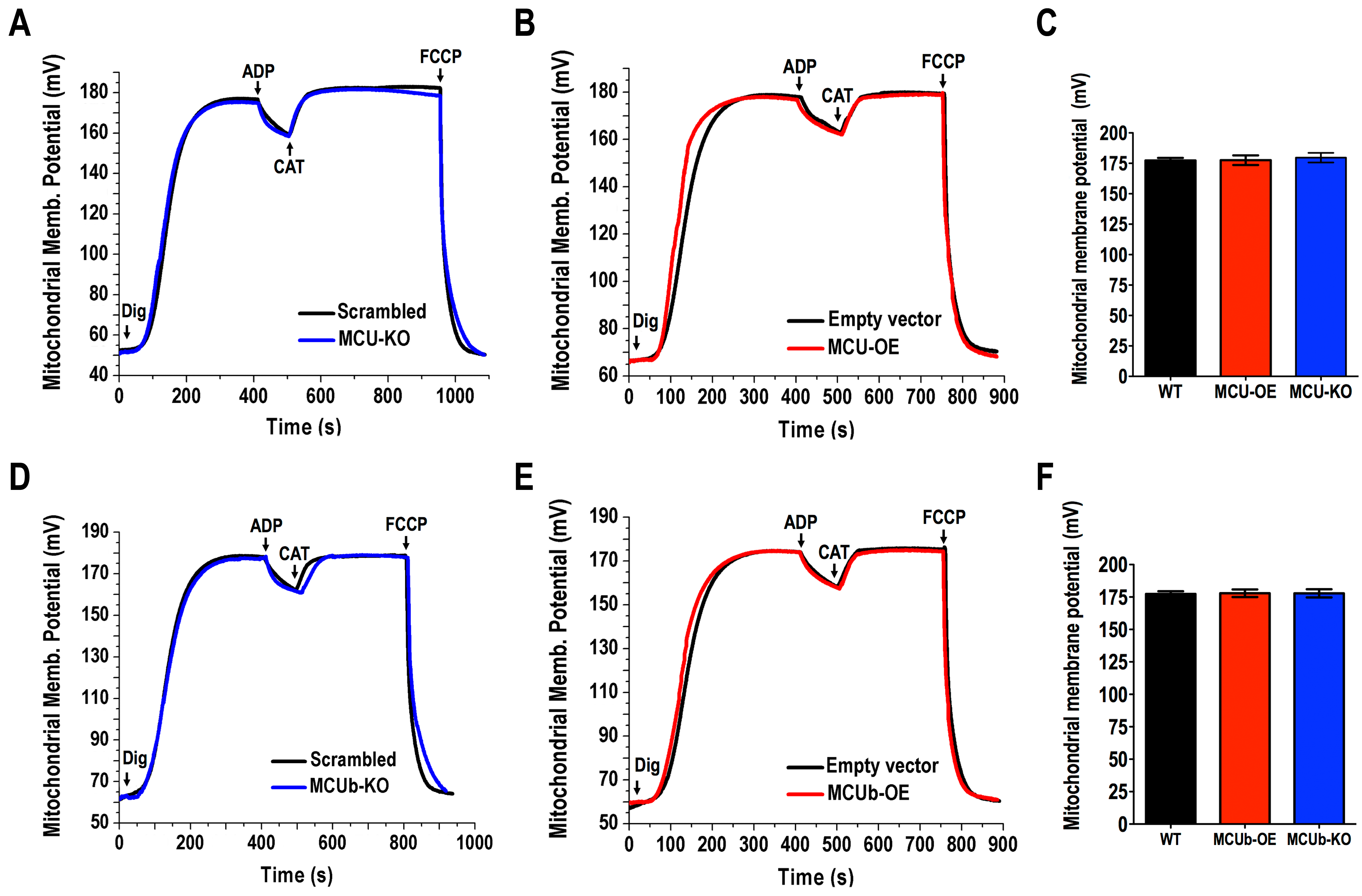

Supplement: FIG S2 [file mbo002173298sf2.tif]

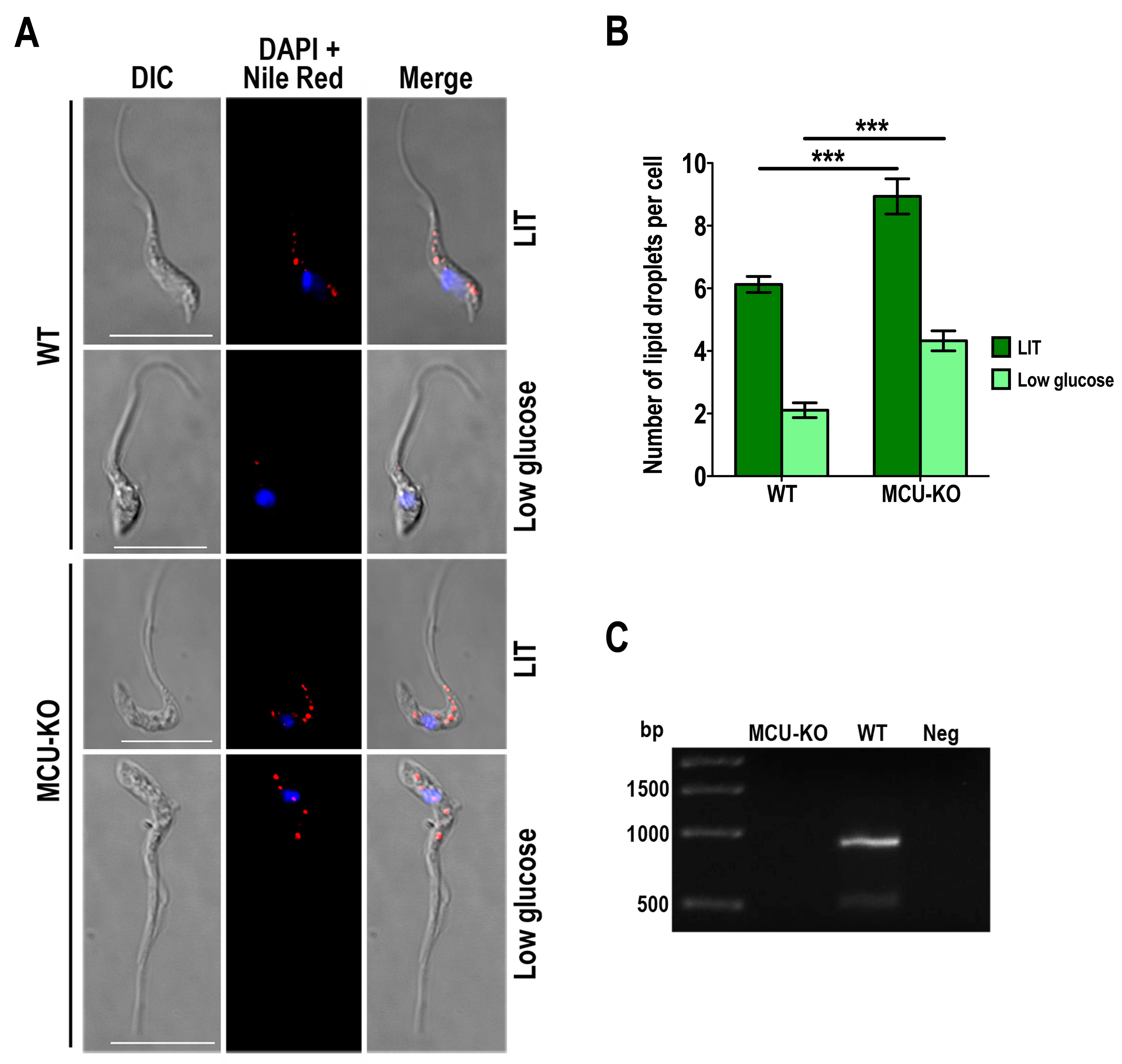

Supplement: FIG S3 [file mbo002173298sf3.tif]

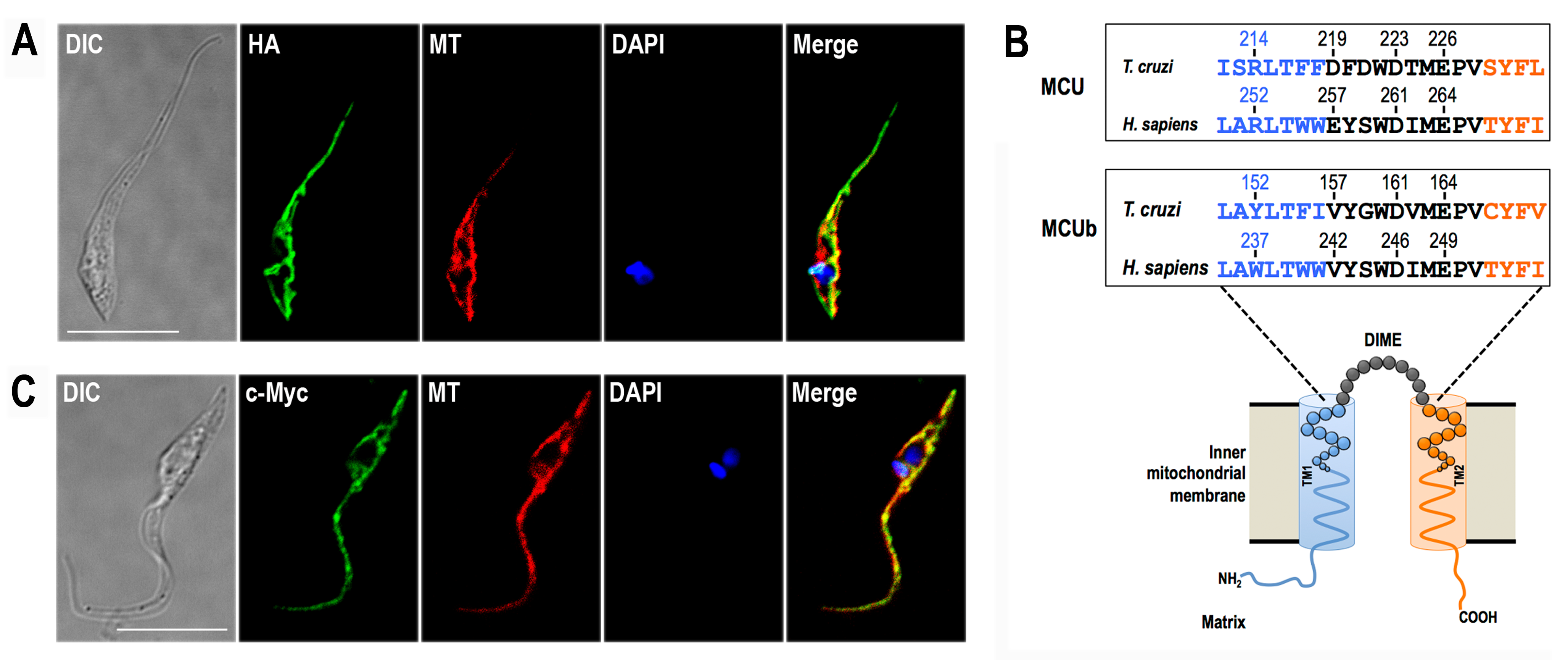

Supplement: FIG S4 [file mbo002173298sf4.tif]

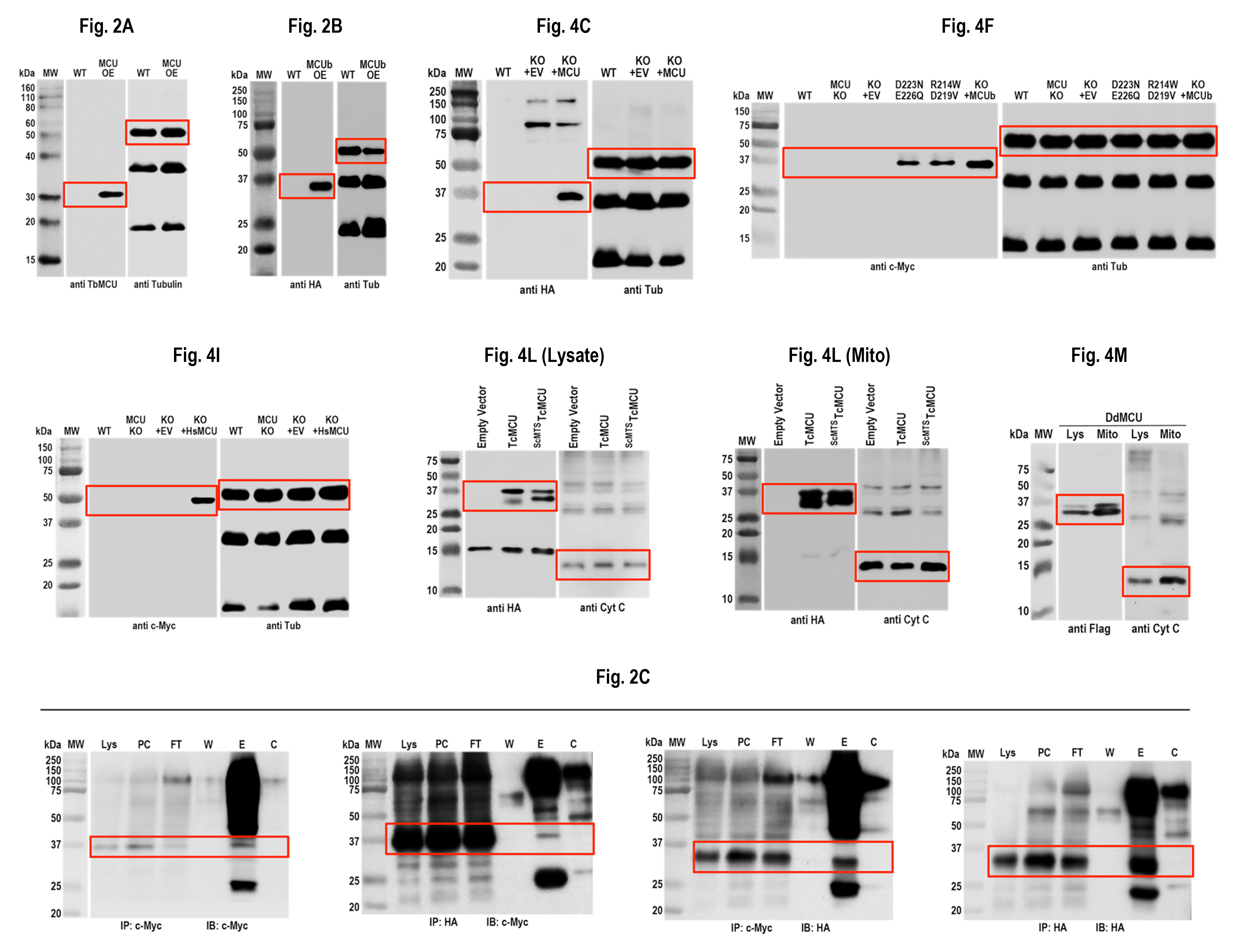

Supplement: FIG S5 [file mbo002173298sf5.tif]
