# Supplementary material for: Different Roles of Mitochondrial Calcium Uniporter Complex Subunits in Growth and Infectivity of Trypanosoma cruzi
Source: mBio. 2017 May 9;8(3):e00574-17. doi: 10.1128/mBio.00574-17 (PMC5424207; doi:10.1128/mBio.00574-17)
Supplement: TEXT S1 [file mbo002173298s1.docx]

**Supplemental information for**

**Different roles of mitochondrial calcium uniporter complex subunits in growth and infectivity of *Trypanosoma cruzi***

Miguel A. Chiurillo^1,*^, Noelia Lander^1^, Mayara S. Bertolini^1^, Melissa Storey^2^, Anibal E. Vercesi^1^, and Roberto Docampo^1,2,*^

^1^Departamento de Patología Clínica, Universidade Estadual de Campinas, Campinas, São Paulo 13083, Brazil and ^2^Center for Tropical and Emerging Global Diseases and Department of Cellular Biology, University of Georgia, Athens, Georgia 30602, USA

^@^To whom orrespondence should be addressed Email: [mchiurillo@yahoo.com](mailto:mchiurillo@yahoo.com) or [rdocampo@uga.edu](mailto:rdocampo@uga.edu)

**Supplemental Material and Methods**

**Supplemental Figures S1 to S5**

**Supplemental Table S1Additional Material and Methods**

**Reagents.** Rabbit polyclonal antibody against TcATG8.1 was a gift from Dr. Vanina Alvarez (Universidad Nacional de San Martin, Argentina). Rabbit polyclonal antibody against TbVDAC was a gift from Dr. Minu Chaudhuri (Meharry Medical College, TN). Platinum Taq DNA Polymerase High Fidelity, TRIzol reagent, Calcium Green-5N, MitoTracker® Deep Red FM, MitoSOX Red, Hygromycin, blasticidin S-HCl, Alexa-conjugated secondary antibodies, ATP determination kit, Pierce ECL Western blotting substrate, BCA Protein Assay Kit, North2South™ Biotin Random Prime Labeling Kit, North2South™ Chemiluminescent Hybridization and Detection Kit and HA epitope tag monoclonal antibody (clone 2-2.2.14) were from Thermo Fisher Scientific Inc. Rhod-2 AM was from Molecular Probes (Invitrogen). Anti c-Myc monoclonal antibody (clone 9E10) and polyclonal rabbit anti-cytochrome c antibody (H-104) were from Santa Cruz Biotechnology (Dallas, TX). Fluoromount-G® was from SouthernBiotech (Birmingham, AL). [α-^32^P]dCTP (3,000 Ci/mmol) was from PerkinElmer. GoTaq® Flexi DNA Polymerase, Wizard® Plus SV Minipreps DNA Purification System, Wizard® SV Gel and PCR Clean-Up System and Prime-a Gene Labeling System were from Promega. The protein assay reagent, Precision Plus Protein™ Dual Color Standards, Zeta-Probe GT Genomic Testing blotting and nitrocellulose membranes were from Bio-Rad. The primers were purchased from Exxtend (Campinas, Brazil). Carboxyatractyloside (CAT), oligomycin, antimycin A_1_, Safranine O, carbonyl cyanide 4-(trifluoromethoxy)phenylhydrazone (FCCP), Benzonase® nuclease, polyclonal rabbit anti Flag antibody and all other reagents of analytical grade were from Sigma.

**Reconstitution of the mitochondrial calcium uniporter in yeast.** *D. discoideum* *MCU* (*DdMCU*), *TcMCU*, and *^ScMTS^TcMCU* chimera [mitochondrial targeting sequence of 74 nt of yeast cytochrome c oxidase subunit IV (ScCox4p) + 819 nt of *TcMCU*] were cloned into the HindIII sites of pACT2 (lacking the GAL4-HA activation domain) using the In-Fusion Cloning Kit (Clontech) (Table S1, primers 28 to 30). The GAL4 activation domain was retained as a negative control in the empty vector. Plasmids were introduced into the yeast strain W303 (MATa ade2-1 his3-11,15 leu2-3,112 ura3, trp1-1, can1- 100) using the lithium acetate transformation method(1). Cells were grown in synthetic dropout medium lacking leucine. *DdMCU* (XM_632658 codon optimized for human expression) and the yeast strain were kindly provided by Dr. Vamsi Mootha (2). Yeast transformants were analyzed by PCR to verify the presence of molecular constructs.

**Spheroblast preparation and measurements.** We followed the methodology reported by Kowaltowski et al. (3) with minor modifications. Cells were harvested by centrifugation (10 min at 1,000 x g), weighed and suspended in 3 ml/g (wet weight) of 1 M sorbitol, 50 mM Tris buffer pH 7.5, 10 mM MgCl_2_, and 30 mM dithiothreitol (DTT). After 15 min incubation at 30°C, the suspension was centrifuged, and the resulting pellets were resuspended in 5 ml/g of 1 M sorbitol, 50 mM Tris buffer pH 7.5, 10 mM MgCl_2_, 1 mM DTT, containing 20U/g of Zymolyase (Zymo Research). The suspensions were incubated at 30°C for 60 min, until conversion to spheroblasts was observed, as assessed by osmotic swelling after suspension in deionized water and determination of absorbance at 600 nm. The spheroblast suspension was then centrifuged and washed twice with 1 M sorbitol, 50 mM Tris buffer pH 7.5 and 10 mM MgCl_2._ The final pellet was suspended to a concentration of approximately 100 mg/ml in the same buffer, and kept on ice. Calcium uptake analysis was conducted at 28°C in a standard reaction medium containing 125 mM sucrose, 65 mM KCl, 10 mM Hepes buffer (pH 7.2), 0.2% BSA, 5 mM succinate, 5 mM malate, 5 mM pyruvate, 5 mM α-ketoglutarate and 1 mM glutamate. Mitochondrial fractions were prepared as described previously (4).

**Sequence analysis.** All molecular constructs were verified by sequencing at the LaCTAD facility (http://www.lactad.unicamp.br). Primers design and sequence analysis were carried out using DNAMAN software (version 7.212, Lynnon Corp., Canada).

**Cell Transfections***. T. cruzi* epimastigotes (4 x 10^7^ cells), were washed with PBS, pH 7.4, at room temperature and transfected in ice-cold cytomix (25 mM Hepes, 120 mM KCl, 0.15 mM CaCl_2_, 10 mM K_2_HPO_4_, 2 mM EDTA, 5 mM MgCl_2_, pH 7.6), containing 25 μg of each DNA construct in 4 mm electroporation cuvettes with 3 pulses (1,500 V, 25 µF) delivered by a Gene Pulser Xcell (Bio-Rad). Stable cell lines were established and maintained under drug selection with appropriate antibiotic(s) (250 μg/ml G418, 10 μg/ml blasticidin, 350 μg/ml hygromycin, 5 μg/ml puromycin). Transfectant parasites were cultured in LIT media supplemented with 20% heat-inactivated FBS until obtaining stable cell lines.

**Western blot analysis.** Western blot analyses were performed using standard procedures (5). Parasites were washed twice in PBS and resuspended in radio-immunoprecipitation assay buffer (RIPA: 150 mM NaCl, 20 mM Tris-HCl [pH 7.5], 1 mM EDTA, 1% SDS, 0.1% Triton X-100) plus a mammalian cell protease inhibitor mixture (Sigma P8340, diluted 1:250), 1 mM phenylmethylsulfonyl fluoride, 2.5 mM tosyl phenylalanyl chloromethyl ketone (TPCK), 100 μM N-(trans-epoxysuccinyl)-L-leucine 4-guanidinobutylamide (E64), and Benzonase nuclease (25 U/ml of culture). Then cells were incubated for 1 h on ice. Cell lysis was verified under a light microscope, and protein concentration was determined by Pierce BCA protein assay. Thirty micrograms of protein from each cell lysate was mixed with 6X Laemmli sample buffer and analyzed by SDS-PAGE in 12% gels. Separated proteins were transferred onto nitrocellulose membranes (Bio-Rad) with a Bio-Rad Trans-blot apparatus. Membranes were blocked with 5% nonfat dried skim milk in PBST (PBS containing 0.1% [vol/vol] Tween 20) overnight at 4°C. Next, blots were incubated for 1 h at RT with a primary antibody, i.e. mAb anti HA-Tag (1:5,000), mAb anti tubulin (1:40,000), mAb anti-c-Myc-Tag (1:00), guinea pig anti-TbMCU (1:500), rabbit anti-cytochrome c (1:100). After three washes with PBST, blots were incubated with the secondary antibody (goat anti-mouse IgG, HRP-conjugated antibody, diluted 1:10,000 or goat anti- rabbit IgG, HRP-conjugated antibody, diluted 1:10,000). Membranes were washed three times with PBST and incubated with Pierce ECL western blotting substrate according to the manufacturer’s instructions. Western blot images were obtained and processed with a C-DiGit Blot Scanner (LI-COR Biosciences).

**Immunoprecipitation.** For co-immunoprecipitation analysis of TcMCU and TcMCUb we obtained a stable epimastigote population expressing both proteins after cotransfecting them with pTREX-h-*TcMCU*-3xc-Myc and pTREX-n-*TcMCUb*-3xHA plasmids. pTREX-h-*TcMCU*-3xc-Myc was obtained by cloning a PCR fragment containing the full sequence of *TcMCU* (Table S1, primers 20 and 21) into pTREX-h-3xc-Myc vector by XbaI and XhoI restrictions sites. pTREX-n-*TcMCUb*-3xHA was obtained as described above. Two independent immunoprecipitations were performed with total protein extracts from this cell line and anti-c-Myc antibody (mAb clone 9E10) or anti-HA antibody (mAb clone 2-2.2.14), using the Pierce Crosslink Immunoprecipitation kit and following manufacturer’s instructions, but skipping the crosslinking step. Briefly, 10 µg of antibody diluted in 1X coupling buffer were bound to Protein A/G plus agarose on a mixer for 1 h at room temperature, washed twice with 1X Coupling Buffer and incubated with 10 mg of pre-cleared lysate diluted in IP Lysis/Wash Buffer overnight at 4°C with gentle end-over-end mixing. A control agarose resin was separately incubated with each antibody as negative controls of the assay. Next, each resin was washed 3 times with 200 µl IP Lysis/Wash Buffer and once with 100 µl 1X Conditioned Buffer. Antigens were eluted with 50 ul Elution Buffer by centrifugation in 1.5 collection tubes containing 5 µl of 1M Tris-HCl, pH 9.5 for immediate neutralization. As crosslinking step was omitted, the antibody (light and heavy immunoglobulin chains) co-eluted with the antigens. 25-µl of each eluted fraction were loaded into 12% acrylamide gels together with different fractions collected during the immunoprecipitation process: total lysate, pre-cleared lysate, flow through and last wash, for western blot analyses with anti-c-Myc and anti-HA antibodies as described above.

**Southern blot analysis.** For Southern blot analysis of *TcMCU*-KO cells, total genomic DNA was isolated from epimastigotes by phenol-chloroform extraction, digested with SphI, separated on a 0.8% agarose gel, and transferred to nylon membranes. The blot was hybridized with a radiolabeled fragment of 100 bp (*TcMCU*: +240 to +340 nt) obtained by PCR (Table S1, primers 13 and 14) using cloned *TcMCU* gene as template. Probe was labeled using random hexanucleotide primers and the Klenow fragment of DNA polymerase I (Prime-a-Gene labeling system, Promega). Hybridizations were carried out in 0.5 M Na_2_HPO_4_, pH 7.2, 7% SDS, 1 mM EDTA and 1X [Denhardt's Solution](https://www.thermofisher.com/order/catalog/product/750018) at 65°C for 18 h. After hybridization, filters were washed with 0.1X SSC, 0.1% SDS solution twice at 65°C for 15 min. Membrane were exposed to films for 24–72 h at −80°C and developed in a dark room.

Alternatively, to check *TcMCUb*-KO by Southern blot, approximately 25 µg of gDNA were digested with MspI enzyme and resolved on a 0.8% agarose gel. Restriction fragments were transferred to nylon membrane and hybridized with a biotin-labeled probe which spams the ~430 nt of *TcMCUb* gene replaced with the *Bsd* gene. Probe for *TcMCUb* was produced via PCR using primers 36 and 37 (Table S1) and labeled using the North2South™ Biotin Random Prime Labeling Kit (Thermo Scientific). Hybridization, post hybridization washes and detection were conducted with North2South™ Chemiluminescent Hybridization and Detection Kit (Thermo Scientific) according manufacture’s recommendations. Signal detection was performed using UVItec Alliance Gel Documentation System (UVItec, Cambridge, UK).

**Immunofluorescence microscopy.** Epimastigotes were washed with PBS and fixed with 4% paraformaldehyde in PBS for 1 h at RT. Cells were allowed to adhere to poly-L-lysine-coated coverslips and then permeabilized for 5 min with 0.1% Triton X-100. Then cells were blocked overnight at 4°C with PBS containing 3% BSA, 1% fish gelatin, 50 mM NH_4_Cl, and 5% goat serum. Next, cells were incubated with a primary antibody (guinea pig polyclonal anti-TbMCU [1:50], rabbit polyclonal anti-TbVDAC [1:200], monoclonal anti-HA [1:5000], monoclonal anti-c-Myc [1:10] or rabbit anti-TcATG8.1 [1:100]), diluted in 1% BSA in PBS (pH 8.0) for 1 h at RT. Excess primary antibody was removed from the cells with three washes with 1% BSA in PBS (pH 8.0), and then cells were incubated for 1 h at RT in the dark with an Alexa Fluor 488-conjugated goat anti-mouse or Alexa Fluor 546-conjugated goat anti-rabbit secondary antibodies (1:1,1000). Following incubation with the secondary antibody, cells were washed and mounted on slides. DAPI (5 µg/ml) was included in the mounting medium to stain DNA. Secondary-antibody controls were performed as described above but in the absence of a primary antibody. Differential interference contrast and fluorescence optical images were captured under nonsaturating conditions with a confocal microscope Leica TCS SP5 II, with a 100x objective (1.44 aperture), that uses photomultiplier tubes for detection of emission, and LAS AF software (Leica, Wezlar, Germany) for acquisition and processing of digital images.

**Ca^2+^ uptake by digitonin-permeabilized *T. cruzi* epimastigotes.** The uptake of Ca^2+^ by permeabilized *T. cruzi* epimastigotes was assayed by fluorescence measurements at 28°C using Calcium Green-5N. Cells were collected by centrifugation at 1,000 x *g* for 7 min and washed twice with buffer A with glucose (BAG, 116 mM NaCl, 5.4 mM KCl, 0.8 mM MgSO_4_, 5.5 mM D-glucose and 50 mM HEPES at pH 7.0). Epimastigotes were resuspended to a final density of 1 x 10^9^ cells/ml in BAG and kept on ice. Before each experiment a 50-μl aliquot of *T. cruzi* epimastigotes (5 x 10^7^ cells) in cell suspension was added to the reaction buffer (125 mM sucrose, 65 mM KCl, 10 mM Hepes–KOH buffer, pH 7.2, 1 mM MgCl_2_, 2.5 mM potassium phosphate; 1.95 ml) containing 0.5 μM Calcium Green-5N. Ca^2+^ uptake by the cells was initiated by the addition of 50 mM digitonin. Fluorescence changes were monitored in a F-7000 fluorescence spectrophotometer (Hitachi) with excitation at 506 nm and emission at 532 nm.

**Assessment of mitochondrial membrane potential.** Estimation of mitochondrial membrane potential in situ was done spectrofluorometrically using the indicator dye Safranine O(6). *T. cruzi* epimastigotes (5 x 10^7^ cells) were incubated at 28°C in reaction buffer (125 mM sucrose, 65 mM KCl, 10 mM Hepes–KOH buffer, pH 7.2, 1 mM MgCl_2_, 2.5 mM potassium phosphate; 1.95 ml) containing 2 mM succinate, 0.2% BSA, 50 μM EGTA and 5 mM safranine, and the reaction was started with digitonin (50 μM). ADP (250 μM), carboxyatractyloside (20 µM), CaCl_2_ (50 μM), EGTA (500 μM) and FCCP (4 μM) were added to the media at different time points. Fluorescence changes were monitored on a Hitachi 7000 spectrofluorometer (excitation = 495 nm; emission = 586 nm). Calibration of ΔΨ_m_ was done as reported before (7).

**Mitochondrial Ca^2+^ concentration.** *T. cruzi* epimastigotes were collected by centrifugation and suspended in 5 ml buffer A with glucose containing 2 µM Rhod-2 AM and 0.02% Pluronic F127. After incubation for 60 min in the dark with gentle shaking, cells loaded with Rhod-2 dye were washed and resuspended in buffer A with glucose to a final density of 1 x 10^9^ cells per ml and kept on ice. The mitochondrial Ca^2+^ concentration was monitored on a Hitachi 7000 spectrofluorometer with excitation at 552 nm and emission at 581 nm.

**Oxygen uptake.** The OCR of digitonin-permeabilized epimastigotes was measured using a high-resolution respirometer (Oroboros Oxygraph-2k, Innsbruck, Austria) with DatLab 4 software for data acquisition and analysis, and calibrated as reported by their manufacturers. Cells (1 x 10^8^) were incubated at 28ºC in a 2 ml chamber containing 125 mM sucrose, 65 mM KCl, 10 mM Hepes-KOH, pH 7.2, 2.5 mM K_2_PO_4_, 1 mM MgCl_2_, 50 μM EGTA, 5 mM succinate and 25 µM digitonin. OCR was calculated as the negative time derivative of oxygen concentration measured in the closed respirometer chambers and expressed per mg of protein. Data were recorded at 2 s intervals, and 10 data points were used to calculate the slope of the OCR plot through a polynomial fit with DataLab 4 software, as described (8).

**Mitochondrial mass.** MitoTracker® Deep Red FM was used for estimation of the mitochondrial mass of *T. cruzi* epimastigotes. Briefly, cells were incubated with 100 nM MitoTracker for 30 min at 28°C in culture medium, then washed with PBS and fixed with 4% paraformaldehyde in PBS for 1 h at RT. Next, cells were allowed to adhere to poly-L-lysine-coated coverslips, washed with PBS and incubated for 1 h at RT with 100 mM NH_4_Cl in PBS, washed again and mounted on slides with Fluoromount G containing 5 µg/ml DAPI. Differential interference contrast and fluorescence optical images were captured under nonsaturating conditions with a confocal microscope Leica TCS SP5 II, with a 100x objective (1.44 aperture). Fluorescence intensity (RFU) of at least 200 cells was quantified using LAS AF Lite software (Leica, Wezlar, Germany). Mitochondrial mass was also estimated by measuring citrate synthase activity in *T. cruzi* epimastigotes using a previously described protocol (9) adapted to trypanosomes. Briefly, the conversion of oxaloacetate and acetyl-CoA to citrate and SH-CoA was monitored by measuring the colorimetric product thionitrobenzoic acid(10). *T. cruzi* epimastigotes in early exponential phase (~1 x 10^8^ cells) were washed twice with PBS and incubated in lysis buffer (10 mM Tris-HCl, pH 7.4, 1 mM EDTA, 0.1% Triton X-100 and 25 units of Benzonase nuclease) for 10 min on ice. Then, proteins were quantified by BCA protein assay and 260 µl reactions were set up in buffer containing 5 µg protein, 250 μM oxaloacetate, 50 μM acetyl-CoA, 100 μM 5,5’-dithio-bis(2-nitrobenzoic acid) and 10 mM Tris-HCl, pH 8.0. The increase in absorbance at 412 nm was monitored for 20 min at 28ºC using a microplate reader (PowerWave XS 2, BioTek Instruments, Winooski, VT, USA). Values of Vmax were normalized taking wild type cell line as reference value.

**Mitochondrial oxidative stress.** Mitochondrial ROS production was measured during the exposure of *T. cruzi* epimastigotes to digitonin in the presence or absence of Ca^2+^ using the fluorescent, mitochondrially targeted probe MitoSOX Red (Invitrogen) as described (11) with some modifications. Epimastigotes (2.5 x 10^8^ cells per ml) were loaded with 5 mM MitoSOX Red in buffer A with glucose for 10 min at 28 °C and washed once with the same buffer. Cells were resuspended to a final density of 1 x 10^9^ cells per ml in the buffer A with glucose and kept on ice. A 100-µl aliquot (1.0 x 10^8^ cells) of the cell suspension was added to the reaction buffer with or without 0.5 mM Ca^2+^ and the reagents indicated in the figure legends. The reaction was initiated by the addition of 50 μM digitonin. Fluorescence changes were monitored in a fluorometer with excitation at 510 nm and emission at 580 nm.

**Autophagy assay.** Expression of the TcAtg8.1 autophagy marker and autophagosome formation in *T. cruzi* epimastigotes grown in LIT medium and under starvation conditions was estimated by immunofluorescence analyses using anti-TcATG8.1 antibody as described (12). For starvation induction, mid-log parasites were washed twice with PBS, resuspended in the same buffer at a concentration of 5 x 10^7^ cells/ml, and incubated for 16 h at 28 °C as described previously (12).

**Adenine nucleotide levels.** Wild type, *TcMCU*-KO and *TcMCUb*-KO epimastigotes were harvested and washed once with buffer A (116 mM NaCl, 5.4 mM KCl, 0.8 mM MgSO_4_ and 50 mM HEPES at pH 7.0). After washing, 1 x 10^8^ cells per treatment were centrifuged and resuspended in 100 μl of buffer A, and then lysed on ice for 30 min by addition of 150 μl of 0.5 M HClO_4_. The lysates were neutralized (pH 6.5) by addition of 60 μl of 0.72 M KOH/0.6 M KHCO_3_. Samples were centrifuged at 1000 x *g* for 5 min and the supernatant was separated for adenine nucleotide determination. ATP, ADP and AMP in extracted samples were quantified by a luciferin–luciferase bioluminescence assay in a luminometer as described (13, 14) with some modifications. We used an ATP Determination Kit (Invitrogen) according to the manufacturer’s instructions with adenylate kinase and/or nucleoside-diphosphate kinase (NDK; Sigma). To determine the amount of adenine nucleotides, four measurements were taken of three different reactions for each sample by end point determination of the ATP concentration: one reaction without addition of any ATP-generating enzyme (for ATP), another reaction adding NDK (for ATP + ADP) and the third reaction adding both adenylate kinase and NDK (for ATP + ADP + AMP). The amount of ADP was obtained by subtracting the ATP value from the (ATP + ADP) value and the amount of AMP was calculated from the difference between the (ATP + ADP + AMP) content and the (ATP + ADP) content.

**REFERENCES**

1. **Gietz RD, Woods RA.** 2002. Transformation of yeast by lithium acetate/single-stranded carrier DNA/polyethylene glycol method. Methods Enzymol 350:87-96.

2. **Kovacs-Bogdan E, Sancak Y, Kamer KJ, Plovanich M, Jambhekar A, Huber RJ, Myre MA, Blower MD, Mootha VK.** 2014. Reconstitution of the mitochondrial calcium uniporter in yeast. Proc Natl Acad Sci U S A 111:8985-8990.

3. **Kowaltowski AJ, Vercesi AE, Rhee SG, Netto LE.** 2000. Catalases and thioredoxin peroxidase protect Saccharomyces cerevisiae against Ca^2+^-induced mitochondrial membrane permeabilization and cell death. FEBS Lett 473:177-182.

4. **Jarmuszkiewicz W, Milani G, Fortes F, Schreiber AZ, Sluse FE, Vercesi AE.** 2000. First evidence and characterization of an uncoupling protein in fungi kingdom: CpUCP of Candida parapsilosis. FEBS Lett 467:145-149.

5. **Lander N, Bernal C, Diez N, Anez N, Docampo R, Ramirez JL.** 2010. Localization and developmental regulation of a dispersed gene family 1 protein in *Trypanosoma cruzi*. Infect Immun 78:231-240.

6. **Figueira TR, Melo DR, Vercesi AE, Castilho RF.** 2012. Safranine as a fluorescent probe for the evaluation of mitochondrial membrane potential in isolated organelles and permeabilized cells. Methods Mol Biol 810:103-117.

7. **Vercesi AE, Bernardes CF, Hoffmann ME, Gadelha FR, Docampo R.** 1991. Digitonin permeabilization does not affect mitochondrial function and allows the determination of the mitochondrial membrane potential of *Trypanosoma cruzi* in situ. J Biol Chem 266:14431-14434.

8. **Pesta D, Gnaiger E.** 2012. High-resolution respirometry: OXPHOS protocols for human cells and permeabilized fibers from small biopsies of human muscle. Methods Mol Biol 810:25-58.

9. **Figueira TR, Castilho RF, Saito A, Oliveira HC, Vercesi AE.** 2011. The higher susceptibility of congenital analbuminemic rats to Ca^2+^-induced mitochondrial permeability transition is associated with the increased expression of cyclophilin D and nitrosothiol depletion. Mol Genet Metab 104:521-528.

10. **Shepherd D, Garland PB.** 1969. The kinetic properties of citrate synthase from rat liver mitochondria. Biochem J 114:597-610.

11. **Irigoin F, Inada NM, Fernandes MP, Piacenza L, Gadelha FR, Vercesi AE, Radi R.** 2009. Mitochondrial calcium overload triggers complement-dependent superoxide-mediated programmed cell death in *Trypanosoma cruzi*. Biochem J 418:595-604.

12. **Alvarez VE, Kosec G, Sant'Anna C, Turk V, Cazzulo JJ, Turk B.** 2008. Autophagy is involved in nutritional stress response and differentiation in *Trypanosoma cruzi*. J Biol Chem 283:3454-3464.

13. **Jansson V, Jansson K.** 2003. An enzymatic cycling assay for adenosine 5'-monophosphate using adenylate kinase, nucleoside-diphosphate kinase, and firefly luciferase. Anal Biochem 321:263-265.

14. **Spielmann H, Jacob-Muller U, Schulz P.** 1981. Simple assay of 0.1-1.0 pmol of ATP, ADP, and AMP in single somatic cells using purified luciferin luciferase. Anal Biochem 113:172-178.

15. **Lander N, Chiurillo MA, Storey M, Vercesi AE, Docampo R.** 2016. CRISPR/Cas9-mediated endogenous C-terminal tagging of *Trypanosoma cruzi* genes reveals the acidocalcisome localization of the inositol 1,4,5-trisphosphate receptor. J Biol Chem 291:25505-25515.
